# Poststroke Cardiorespiratory Exercise for Brain Volume and Cognition: A Randomized Clinical Trial

**DOI:** 10.1001/jamanetworkopen.2025.28907

**Published:** 2025-08-26

**Authors:** Amy Brodtmann, Leonid Churilov, Kimberley Adkins, Ruwayda Haibe, Stephanie Tucker, Mohamed Salah Khlif, Emilio Werden, Laura J. E. McCambridge, Rachael Telfer, Sharon Kramer, Barbara R. Cardoso, Matthew Pase, Nathalie Launder, Natalia Egorova-Brumley, Stanley Hughwa Hung, Louise M. Burrell, Gavin Williams, Vincent Thijs, Julie Bernhardt, Liam Johnson, Kathryn S. Hayward, PISCES-ZODIAC Investigators

**Affiliations:** 1 Department of Neuroscience, School of Translational Medicine, Monash University, Melbourne, Australia; 2 Department of Medicine, University of Melbourne, Melbourne, Australia; 3 The Florey Institute for Neuroscience and Mental Health, Melbourne, Australia; 4 Department of Nutrition, Dietetics and Food, Monash University, Melbourne, Australia; 5 Victorian Heart Institute, Monash University, Melbourne, Australia; 6 Turner Institute, Monash University, Melbourne, Australia; 7 Melbourne School of Psychological Sciences, University of Melbourne, Melbourne, Australia; 8 Department of Physical Therapy, Faculty of Medicine, University of British Columbia, Vancouver, Canada; 9 Austin Health, Melbourne, Australia; 10 Department of Physiotherapy, University of Melbourne, Melbourne, Australia; 11 Physiotherapy Department, Epworth HealthCare, Melbourne, Australia; 12 School of Behavioural and Health Sciences, Australian Catholic University, Melbourne, Australia

## Abstract

**Importance:**

Stroke increases the risk of cognitive impairment and dementia without proven prevention therapies. Cardiorespiratory exercise (CRX) preserves brain health.

**Objective:**

To determine whether a CRX intervention preserves hippocampal volume (HV) and cognition in patients after ischemic stroke.

**Design, Setting, and Participants:**

The Post-Ischemic Stroke Cardiovascular Exercise Study (PISCES)–Zoom Delivered Intervention Against Cognitive Decline (ZODIAC) is a phase 2b assessor-blinded randomized clinical trial performed at 4 metropolitan health care services in Melbourne, Australia. Eligible participants included adult patients who survived ischemic stroke without comorbidities prohibiting exercise or diagnosed cognitive disorder. Participants were recruited from May 26, 2016, to March 20, 2020, for PISCES in-person training and from November 9, 2020, to February 12, 2024, for ZODIAC remotely delivered home training. A total of 6921 participants were screened for eligibility, 130 were recruited, 107 were randomized (34 in PISCES and 73 in ZODIAC), and 104 continued to intervention. Study visits at 2, 4, and 12 months post stroke included brain magnetic resonance imaging and cognitive testing. Participants were randomized (1:1), stratified by baseline function (modified Rankin Scale score of 0-1 vs 2-3) and total brain volume. Primary, secondary efficacy, and safety outcome analyses were conducted using modified intention-to-treat (mITT) principle and per protocol.

**Intervention:**

Participants received 8 weeks of three 60-minute sessions/wk. Participants in the CRX group received prescribed intensity progressive aerobic and resistance training; the control group received balance and stretching training.

**Main Outcomes and Measures:**

The primary outcome was relative change in HV calculated as the difference between HV at times 1 and 2 divided by HV at time 1. Secondary outcome consisted of 12-month executive function test results (Trial Making Test, Part B [TMT-B]), adjusted for baseline TMT-B and mRS score.

**Results:**

The 104 participants included in primary outcome mITT analysis (55 in the control and 49 in the CRX groups; mean [SD] age, 64 [14] years; 67 [64.4%] male; equivalent baseline mRS score). One hundred participants (33 in PISCES and 67 in ZODIAC) completed 4-month assessments, and 97 (31 in PISCES and 66 in ZODIAC) completed 12-month assessments. There were no intervention-related serious adverse events. Mean (SD) difference in relative change in HV between the CRX (−0.26% [2.12%]) and control (−0.11% [2.35%]) groups was −0.10% (95% CI, −1.10% to 0.87%; *P* = .83). The CRX group (n = 43) performed better than the control group (n = 51) on the adjusted TMT-B at 12 months (adjusted mean difference, −3.75 [95% CI, −5.02 to −2.49] seconds).

**Conclusions and Relevance:**

In this randomized clinical trial of fitness training, CRX was safe but did not preserve HV more than a control condition. CRX may benefit cognitive preservation following ischemic stroke.

**Trial Registration:**

anzcrt.org Identifier: ACTRN12616000942459

## Key Points

**Question:**

Does cardiorespiratory exercise (CRX) preserve brain volume and cognition after stroke?

**Findings:**

In this randomized clinical trial of 104 patients who survived ischemic stroke, an 8-week CRX intervention delivered at 2 months post stroke was safe but did not preserve hippocampal volume more than the balance and stretching control. However, executive and global cognitive performance at 12 months post stroke was better in the CRX group.

**Meaning:**

CRX delivered 2 months after stroke was safe; while it did not preserve hippocampal volume more than the control condition, there was promising evidence of preserved cognition at 12 months after stroke.

## Teaser

This randomized clinical trial assesses the benefits of a cardiorespiratory exercise intervention on hippocampal volume and cognition when delivered to patients at 2 months after ischemic stroke.

## Introduction

Physical inactivity and sedentary behaviors are highly prevalent following stroke.^1^ Cardiorespiratory (aerobic) training after stroke is supported by American Stroke Association^1^ and World Health Organization guidelines,^2^ while noting that the impact on cognitive, mood, and quality of life effects is lacking.^3^ Midlife exercise interventions may preserve brain volume^4,5^ and improve cognitive performance. Regular, lifelong cardiorespiratory exercise (CRX) reduces the risk of all-cause dementia.^6^ CRX is also associated with improvements in cognitive processes dependent on white matter health and hippocampal function and increased total brain^4^ and hippocampal^7^ volumes in older people. Cardiac rehabilitation has been a validated secondary prevention and risk reduction strategy in cardiovascular disease for decades,^8^ yet stroke clinicians lack validated exercise programs to prescribe to their patients. This may be due to lingering concerns about the safety and feasibility of high-intensity exercise interventions.^9^ Clinical trials in patients who survive stroke have not demonstrated improvements in maximal walking speed and Barthel index scores at 3 months post stroke^10^ or in cardiorespiratory fitness and physical activity levels at 12 months.^11^ Cognitive and brain volume effects have not been described.

Metrics of vascular brain health, including white matter hyperintensities^12^ and hippocampal atrophy, are strongly associated with both functional and cognitive impairment.^13,14^ Focal brain atrophy precedes and predicts cognitive impairment in the most common form of dementia, Alzheimer dementia.^15^ Similarly, early poststroke cognitive impairment is associated with greater brain atrophy^16^ and the risk of poststroke dementia.^17^ General and focal brain atrophy rates^18^ are increased in the months after stroke compared with stroke-free controls^19^ and are associated with cognitive impairment.^16^ The hippocampi appear especially vulnerable to rapid poststroke atrophy,^18,19^ demonstrated by rapid volume loss (0.64 mm^3^/d, or 1.25% atrophy) during the first 3 months after stroke.^19^ Conversely, hippocampal volume (HV) increases after aerobic exercise intervention have been demonstrated in stroke-free middle-aged people^20,21^ and people with Alzheimer dementia.^22^ Patients who survive ischemic stroke who spent more time in moderate to vigorous physical activity had reduced white matter hyperintensity volume compared with less active peers,^23^ and those who spent more time active had better attention performance and increased network connectivity in the dorsal attention network.^24^ These changes in imaging metrics and cognitive performance are encouraging evidence for the potential protective effects of physical activity after stroke.

It is possible that exercise interventions in the subacute poststroke period might preserve brain volume and protect against future cognitive impairment. In the Post-Ischemic Stroke Cardiovascular Exercise Study (PISCES)–Zoom Delivered Intervention Against Cognitive Decline (ZODIAC), we assessed the brain benefits of a CRX intervention delivered at 2 months after ischemic stroke. Our primary hypothesis was that a CRX and resistance training intervention would preserve poststroke HV at 4 months greater than a stretch and balance active control intervention (CON). Our secondary aim was to investigate whether the CRX intervention resulted in better cognitive function at 12 months, measured via the Trail Making Test, Part B (TMT-B),^25,26^ adjusted for baseline score and modified Rankin Scale (mRS) score.^27^

## Methods

### Study Procedures

The PISCES-ZODIAC study was a multicenter, prospective, randomized, blinded end point, clinical phase 2b trial.^28,29^ Intervention was delivered in person^29^ until the SARS-CoV-2 pandemic restrictions prompted a change to a home-delivered intervention (CONSERVE [CONSORT and SPIRIT Extension for RCTs Revised in Extenuating Circumstances] checklist and protocol^28^), remotely supervised by our trained exercise professionals.^28^ All study procedures are detailed in published protocols^28,29^ and in Supplement 1. We report findings according to the Consolidated Standards of Reporting Trials (CONSORT) reporting guideline for randomized clinical trials. All patients provided written informed consent.

Participants were recruited from 4 metropolitan university health care services in Melbourne, Australia: Austin, Eastern, Epworth, and Western Health. Central ethics approval was granted by the Austin Health Human Research Ethics Committee, with appropriate site-specific governance approvals. Study visits were conducted at 3 sites.

Adult survivors of ischemic stroke with premorbid mRS scores of 3 or less, English proficiency to understand spoken instructions, and ability to exercise at 2 months post stroke were eligible. Exclusion criteria consisted of no confirmed ischemic stroke, significant medical comorbidities precluding exercise, survival less than 12 months, prestroke cognitive impairment, and contraindication to magnetic resonance imaging (MRI).

Study staff screened participants via hospital discharge summaries or direct notification from clinicians.^28,29^ Initial consent to ascertain MRI eligibility was obtained via video or telephone call or in-person visit. Written consent was provided at the first study visit.

Participants attended 3 study visits^28,29^: before intervention at 2 months post stroke (time 1), following the 8-week exercise intervention at 4 months post stroke (time 2), and at 12 months (time 3). Study procedures at each visit included MRI; functional, cognitive, and mood assessments; and fitness testing. Participants self-reported their gender and ethnicity, cross-checked with hospital records. Sex was reported as assigned at birth.

The randomization schedule was managed by an independent staff member. Randomization occurred following time 1 MRI, stratified by baseline mRS score (0-1 vs 2-3, with higher scores indicating poorer functioning^27^) and baseline total brain volume (TBV)^30^ (low: <1 million mm^3^; high: ≥1 million mm^3^).^28,29^ Adverse events were reported to our medical monitor blinded to study arm. Exercise staff members (K.A., S.T., and R.T.) maintained a separate research electronic data capture database for intervention data entry.

We acquired 3-T MRI scans on scanners at the following sites: 64-channel head-neck coil (Skyra and Prisma-fit; Siemens) at both Melbourne Brain Centre, Florey Institute, and Baker Institute, Alfred Centre, and 8-channel coil (Discovery MR750; GE) at Sunshine Hospital, Western Health (eAppendices 1 and 2 in Supplement 2 provide scanner and sequence details).

Longitudinal FreeSurfer processing pipeline, version 7.3.2, was used to segment 1-mm isotropic T1-weighted images including motion correction, removal of nonbrain tissue, automated Talairach transformation, subcortical white matter and deep gray matter segmentation, intensity normalization, gray matter–white matter boundary tessellation, and automated topology correction. T2-weighted images improved pial surface segmentation. All segmentations were visually inspected and corrected as required.

Cognitive assessments were delivered primarily face to face, with video testing available, including our secondary outcome measure, TMT-B, and a test of global cognition, the Alzheimer Disease Assessment Scale–Cognition subscale (ADAS-Cog^31^). TMT-B is a validated, fully normed^25^ test of attention, processing speed, and executive function.^28,29^ It is a pen-and-paper test measured as time to completion (ie, seconds), with lower scores indicating faster, better executive function, validated as an objective marker of poststroke cognitive recovery.^26^ Global cognitive ability was measured by the ADAS-Cog^31^ at 2 and 12 months.^28^ The ADAS-Cog includes 11 tasks, each of which is scored as a 0 if there is no impairment; hence, lower scores imply better cognition.

Cardiorespiratory fitness testing was performed on a total body recumbent stepper (T5XR; NuStep LLC).^28,29^ A prediction equation based on heart rate was developed in older adults to estimate peak oxygen uptake.^28^

### Intervention

All participants in the CRX group performed a combination of progressive aerobic exercise and resistance training at a prescribed intensity range based on their heart rate reserve for the aerobic exercise and their 3-repetition maximum for the resistance training. Each exercise training session began and ended with a 5-minute warm-up and cool-down at 25% to 35% of the participants’ heart rate reserve. Our active balance and stretching CON intervention was used to ensure participants remained blinded to their allocation; participants consented for an exercise intervention study after stroke.^28,29^

PISCES participants performed center-based CRX on treadmills and gym equipment with moderate intensity resistance training at 70% to 80% of their 3-repetition maximum. ZODIAC participants exercised on upright stationary bicycles (PB10.1-2 and CBSG.2; Steelflex) and did resistance training using free- and body-weight exercises and resistance bands at an intensity that elicited a self-reported rating of 12 to 15 on a Borg Rating of Perceived Exertion (range, 12-15, with higher scores indicating greater perceived exertion).^32^

### Main Outcome Measures

Primary outcome was relative change in HV between the 2- and 4-month visits, defined as (HV[t1] − HV[t2])/HV(t1). Secondary cognitive outcome was TMT-B time in seconds at 12 months, adjusted for baseline time and mRS score. Other secondary outcomes were relative TBV change between 2 and 4 months, measured as (TBV[t1] − TBV[t2])/TBV(t1), and safety outcomes during the intervention period, including number and proportion of participants who died, proportion of participants with at least 1 serious adverse event (SAE) related to the intervention, and proportion of participants with recurrent stroke or transient ischemic attack (TIA) (AE of special interest).

Exploratory clinical outcomes at 12 months included cardiorespiratory fitness, physical activity levels, ADAS-Cog, and mood and functional scales. Exploratory imaging outcomes included relative HV measured as (HV[t1] − HV[t3])/HV(t1) and TBV measured as (TBV[t1] − TBV[t3])/TBV(t1) change between 2 and 12 months. Other exploratory outcomes are listed in the statistical analysis plan (Supplement 1).

### Statistical Analysis

The statistical analysis plan was finalized before database lock and unblinding. We estimated a sample size of 90 participants (45 per group) providing 80% power was needed to detect a difference in HV between arms corresponding to a medium-to-large effect size (Cohen *d* = 0.6), with a 2-tailed *P* ≤ .05 indicating statistical significance. The sample size was inflated by 20% to account for attrition and potential pandemic obstacles,^25^ producing a total sample of 110 participants.

Discrete variables are summarized as frequencies and percentages for baseline characteristics. Unless otherwise indicated, percentages were calculated according to the number of patients for whom data were available. Continuous variables were reported as either mean (SD) or median (IQR), including durations and time intervals.

Outcomes were assessed in all participants under the modified intention-to-treat principle (mITT), that is, all participants who were randomized and received at least 1 intervention session, regardless of available outcome data. Participants who withdrew prior to any study procedures were not included in the mITT analyses but remained in the study database. The per-protocol dataset excluded participants who did not satisfy inclusion or exclusion criteria or did not receive the allocated intervention in the prespecified manner within the prespecified timeframe. Per-protocol eligibility for the intervention dose (total minutes) was defined as participating in 80% or more of the prescribed session number.

All missing data were assumed to be missing at random. For all analyses, complete case analysis was undertaken followed by sensitivity analyses, considering plausible alternative assumptions about missing primary outcome data.

For the primary outcome, we used mixed-effects linear regression models with PISCES and ZODIAC substudies and substudy-by-treatment interaction terms, incorporated as random effects to account for potential differences between PISCES and ZODIAC substudies. Effect estimate was presented as the between-group mean difference in 2- to 4-month relative change in HV with 95% CIs adjusted for baseline HV and functional status (mRS score, 0-1 vs 2-3, with higher scores indicating greater disability). In the prespecified sensitivity analysis for the assumption of missingness at random, data and missingness were modeled jointly using a pattern-mixture model.

For secondary and exploratory HV and TBV measures and cardiorespiratory fitness, we used mixed-effects linear regression models with PISCES and ZODIAC substudies and substudy-by-treatment interaction terms incorporated as random effects as noted above. For cognitive, mood, anxiety, depression, quality of life, and fatigue, we used clustered median regression models with PISCES and ZODIAC substudies as clusters to account for potential differences.

Relevant effect sizes were presented as adjusted difference in medians with respective 95% CIs. SAEs were analyzed using Firth logistic regression models as low incidence of events with effect size presented as an odds ratio. mRS score was analyzed using a mixed-effect ordinal logistic regression model with PISCES and ZODIAC substudies and substudy-by-treatment interaction terms incorporated as random effects to account for potential differences as above. Relevant effect size was presented as an adjusted common odds ratio with respective 95% CI. All analyses (except SAEs due to low event incidence) were adjusted as prespecified (Supplement 1) by baseline relevant score and baseline mRS score. All analyses were conducted on both mITT and per-protocol principles using Stata, version 18.0 SE (StataCorp LLC), and R, version 4.1.3 (R Program for Statistical Computing).

## Results

**Figure 1.**
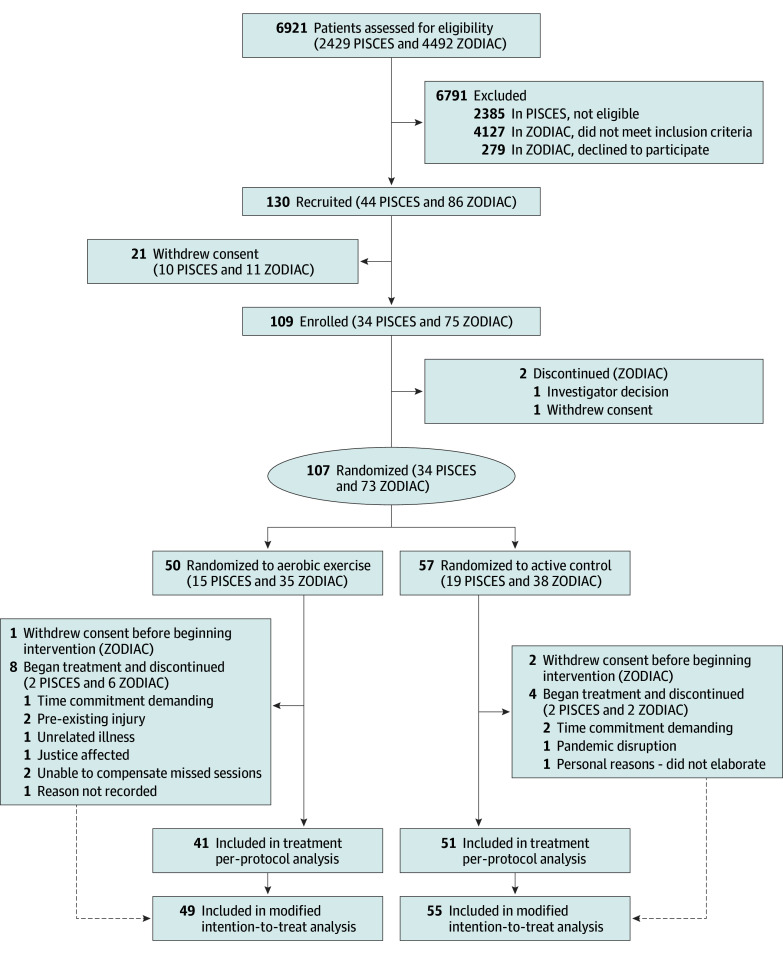
CONSORT Flowchart of Modified Intention-to-Treat Analyses PISCES indicates Post-Ischemic Stroke Cardiovascular Exercise Study; ZODIAC, Zoom Delivered Intervention Against Cognitive Decline.

PISCES (in-person intervention) participants were recruited from May 26, 2016, to March 20, 2020. Recruitment was paused for pandemic restrictions and resumed as ZODIAC (remotely delivered intervention) from November 9, 2020, to February 12, 2024. From 107 randomized participants (34 in PISCES and 73 in ZODIAC), 3 ZODIAC participants discontinued before the intervention started; 104 (55 in the CON group and 49 in the CRX group) were included in the primary outcome mITT analysis. One hundred participants completed the assessment at 4 months (33 in PISCES and 67 in ZODIAC) and 97 at 12 months (31 in PISCES and 66 in ZODIAC) (Figure 1).

Table 1 displays mITT participants’ baseline characteristics (per protocol in eTable 1 in Supplement 2). All participants self-identified as cisgender. Mean (SD) age was 64 (14) years; 37 (35.6%) were female and 67 (64.4%) were male. Participants had a high level of education (median, 15 [IQR, 12-17] years). All baseline variables were well balanced between intervention groups, apart from atrial fibrillation (4 of 46 [8.7%] in the CRX group vs 13 of 51 [25.5%] in the CON group) and type 2 diabetes (11 of 45 [24.4%] in the CRX group vs 6 of 49 [12.2%] in the CON group).

**Table 1.** Participants Included in Modified Intention-To-Treat Analysis

Characteristic	Study arm	
	CRX (n = 49)	CON (n = 55)
Baseline demographic		
Age, median (IQR), y	68.20 (55.81-73.83)	65.96 (56.07-75.44)
Assigned sex at birth, No./total No. (%)^a^		
Male	28/49 (57.1)	39/55 (70.9)
Female	21/49 (42.9)	16/55 (29.1)
Educational level, median (IQR), y	15 (12-16.5)	15 (12-17)
Cardiorespiratory fitness, median (IQR)^b^	15.3 (12.1-24.1)	17 (14.1-24.2)
Modified Rankin scale score, median (IQR)^c^	1 (1-2)	1 (1-2)
National Institute of Health Stroke Scale score, median (IQR)^d^	0 (0-1)	0 (0-1)
First ever clinical stroke, No./total No. (%)	43/49 (87.8)	42/55 (76.4)
Brain MRI		
Baseline hippocampal volume, mean (SD), mm^3^	3571.55 (444.01)	3638.32 (452.06)
Baseline total brain volume, mean (SD), mm^3^	1 107 103 (129 169.9)	1 133 085 (126 053.8)
Baseline stroke lesion volume, median (IQR), mm^3^	1706 (280-6455)	1472 (418-4294)
Vascular risk factors, No./total No. (%)		
Atrial fibrillation	4/46 (8.7)	13/51 (25.5)
Hypertension	27/48 (56.3)	32/55 (58.2)
Dyslipidemia	26/49 (53.1)	34/55 (61.8)
Obesity^e^	13/47 (27.7)	14/52 (26.9)
Type 2 diabetes	11/45 (24.4)	6/49 (12.2)
Smoking history^f^	22/49 (44.9)	31/55 (56.4)
Cognitive status at baseline		
National Adult Reading Test, mean (SD)^g^	108.71 (10.67)	109.48 (9.33)
Montreal Cognitive Assessment, median (IQR)^h^	25 (24-27)	25 (23-27)
Trail Making Test part B, No./total No. (%)^i^	49/49 (100)	55/55 (100)
Time to completion, median (IQR), s^j^	90.0 (61.0-127.8)	82.7 (68.4-137.8)

Abbreviations: CON, control; CRX, cardiorespiratory exercise; MRI, magnetic resonance imaging.

^a^ All participants identified as cisgender to their assigned sex at birth.

^b^ Cardiorespiratory fitness as measured by modified heart rate. Includes 47 CRX and 55 CON participants.

^c^ Higher scores indicate poorer functioning.

^d^ Administered at baseline assessment. Includes 49 CRX and 54 CON participants. Scores range from 0 to 42, with higher scores indicating greater stroke severity.

^e^ Indicates body mass index (calculated as the weight in kilograms divided by the square of the height in meters) greater than 30.

^f^ Smoking status reported as never smoked vs history of smoking/current smoker for our analyses.

^g^ Includes 47 CRX and 53 CON participants. Raw scores are produced as estimated Full Scale Intelligence Quotient (FSIQ). FSIQ has a mean of 100 and a SD of 15. Higher scores indicating higher premorbid FSIQ or ability.

^h^ Scores range from 0 to 30, with higher scores indicating better cognition.

^i^ Higher (longer) times indicating slower (worse) processing speed.

^j^ Includes 49 CRX and 54 CON participants.

Table 2 details the mITT study intervention (per protocol in eTable 2 in Supplement 2). Both groups received an equivalent number of sessions and total time spent with exercise staff per participant at a median of 1185 (IQR, 1053-1329) minutes in the CRX group vs 1225 (IQR, 1168-1440) minutes in the CON group.

**Table 2.** Intervention Summary in Modified Intention-to-Treat Analysis

Exercise intervention	Study arm	
	CRX (n = 49)	CON (n = 55)
Total No. of exercise sessions per participant, median (IQR)	20 (20-23)	21 (20-24)
Total time spent with trained exercise professional per participant, median (IQR), min	1185 (1053-1329)	1225 (1168-1440)
Time spent in CRX training per completed sessions per participant, median (IQR), min	25.0 (23.4-25.9)	NA
Total time spent in CRX training per participant, median (IQR), min	518 (480-574)	NA

Abbreviations: CON, control; CRX, cardiorespiratory exercise; NA, not applicable.

**Figure 2.**
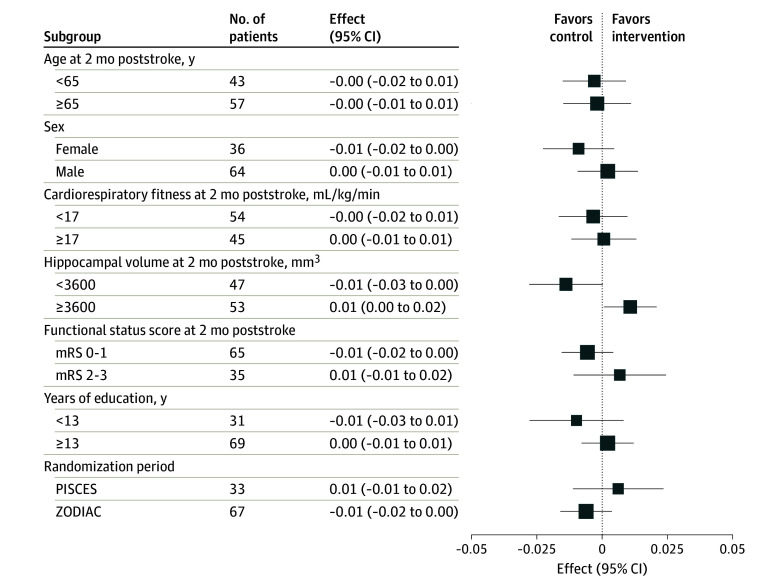
Modified Intention-to-Treat Subgroup Analyses for Change in Hippocampal Volume at 4 Months mRS indicates modified Rankin Scale; PISCES, Post-Ischemic Stroke Cardiovascular Exercise Study; and ZODIAC, Zoom Delivered Intervention Against Cognitive Decline.

Table 3 shows outcomes and adverse events for mITT (per protocol in eTable 3 in Supplement 2). No difference in mean (SD) relative HV change was observed between the CRX (mean [SD], −0.26% [2.12%]) and CON (mean [SD], −0.11% [2.35%]) groups (adjusted mean difference, −0.10%; 95% CI, −1.10% to 0.9%; *P* = .83). We report the mITT sensitivity analysis in eFigure 1 in Supplement 2 (per protocol in eFigure 2 in Supplement 2). No substantive effect modifications were observed on prespecified subgroup analyses for the primary outcome (Figure 2; per protocol in eFigure 3 in Supplement 2). Intervention is summarized in eFigure 4 in Supplement 2. eFigure 5 in Supplement 2 demonstrates stroke lesion map for all study participants.

**Table 3.** Primary, Secondary, and Exploratory Outcomes in Modified Intention to Treat Analysis

Outcome	Study arm		Effect size (95% CI)
	CRX (n = 49)	CON (n = 55)	
Primary			
HV at 4 mo post stroke, mean (SD), mm^3^	3593.84 (417.73)	3630.23 (460.47)	NA
No. of participants	47	53	NA
HV change between 2 and 4 mo post stroke, mean (SD), %^a^	0.26 (2.12)	0.11 (2.35)	−0.10 (−1.10 to 0.87)
No. of participants	47	53	NA
Secondary efficacy			
TBV at 4 mo post stroke, mean (SD), mm^3^	1 112 961 (125 116)	1 127 632 (126 469)	NA
No. of participants	47	53	NA
TBV change between 2 and 4 mo post stroke, mean (SD), %	0.13 (1.10)	0.18 (1.10)	−0.05 (−0.48 to 0.38)
No. of participants	47	53	NA
12-mo TMT-B, No. (%)	43 (87.8)	51 (92.7)	NA
				12-mo TMT-B, median (IQR), s	93 (58 to 130)	88 (58 to 111)	−3.75 (−5.02 to −2.49)
No. of participants	43	51	NA
Safety intervention period 2-4 mo post stroke			
Death, No./total No. (%)	0/49	0/55	NE
SAE, No./total No. (%)	3/49 (6.1)	3/55 (5.5)	0.94 (0.20 to 4.49)^b^
Recurrent stroke or TIA, No./total No. (%)	1/49 (2.0)	1/55 (1.8)	0.002 (−0.05 to 0.06)^c^
Exploratory imaging			
HV at 12 mo, mean (SD), mm^3^	3557.11 (448.75)	3638.17 (453.26)	NA
No. of participants	41	49	NA
HV change between 2 and 12 mo post stroke, mean (SD), %	−0.76 (2.63)	−1.10 (2.10)	−0.15 (−1.10 to 0.81)
No. of participants	41	49	NA
TBV at 12 mo, mean (SD), mm^3^	1 115 461 (121 495)	1 135 112 (121 008)	NA
No. of participants	41	49	NA
TBV change between 2 and 12 mo post stroke, mean (SD), %	−0.68 (1.18)	−0.98 (1.35)	−0.25 (−0.77 to 0.27)
No. of participants	41	49	NA
Exploratory clinical measured at 12 mo			
Cardiorespiratory fitness as peak VO_2_, estimate, mean (SD)	16.57 (5.30)	18.68 (5.25)	−0.25 (−1.50 to 1.01)
No. of participants	37	46	NA
No. of participants with ADAS-Cog, No./total No. (%)	32/49 (65.3)	31/55 (56.4)	NA
ADAS-Cog score, median (IQR)^d^	9.3 (6.7 to 13.4)	10.3 (8.5 to 14.5)	−1.00 (−1.35 to −0.65)
No. of participants	32	31	NA
Modified Rankin Scale score, No. (%)^e^			
0	6/43 (14.0)	11/51 (21.6)	0.52 (0.22 to 1.22)
1	28/43 (65.1)	32/51 (62.7)	
2	7/43 (16.3)	8/51 (15.7)	
3	2/43 (4.7)	0/51	
4	0/43	0/51	
5	0/43	0/51	
6	0/43	0/51	
PASE daily physical activity, median (IQR)^f^	157.9 (92.6 to 182.0)	158.5 (90.6 to 199.9)	−5.10 (−33.82 to 23.62)
No.	40	49	NA
Anxiety GAD-7 score, median (IQR)^g^	2 (0 to 4)	1 (0 to 3)	−0.43 (−1.15 to 0.29)
No.	39	49	
Depression PHQ-9, median (IQR)^h^	5 (1 to 8)	2 (1 to 5)	1.00 (0.71 to 1.29)
No.	38	49	
Quality of life AQoL, median (IQR)^i^	88.89 (80.56 to 94.44)	91.67 (86.11 to 94.44)	−0.93 (−1.74 to −0.11)
No.	40	49	
FAS, median (IQR)^j^	22 (17 to 26)	19 (16 to 22)	−0.38 (−4.96 to 4.19)
No.	39	47	NA

Abbreviations: ADAS-Cog, Alzheimer Disease Assessment Scale–Cognition subscale; AQoL, assessment of quality of life; CON, control; CRX, cardiorespiratory exercise; FAS, Fatigue Assessment Scale; GAD-7, Generalized Anxiety scale-7; HV, hippocampal volume; NA, not applicable; NE, nonestimable due to no events; PASE, Physical Activity Scale for the Elderly; PHQ-9, Patient Health Questionnaire-9; SAE, serious adverse effects; TBV, total brain volume; TIA, transient ischemic attack; TMT-B, Trail Making Test, part B; VO_2_, oxygen uptake.

^a^ *P* = .83.

^b^ Due to only a small number of safety events effect sizes were calculated as odds ratio using Firth logistic regression.

^c^ Due to only a small number of safety events effect sizes were calculated as unadjusted risk difference.

^d^ Scores range from 0 to 70, with higher scores indicating greater cognitive impairment.

^e^ Scores range from 0 to 6, with higher scores indicating worse disability.

^f^ Scores range from 0 to 793, with higher scores indicating greater physical activity.

^g^ Scores of 15 or greater indicate severe anxiety.

^h^ Scores range from 0 to 27, with higher scores indicating more severe depressive symptoms.

^i^ Scores range from 0 to 100, with higher scores indicating better quality of life.

^j^ Scores range from 10 to 50, with higher scores indicating more severe fatigue.

Forty-three of 49 participants (87.8%) in the CRX group and 51 of 55 (92.7%) in the CON group completed the TMT-B at 12 months. Median time was 93 (IQR, 58-130) seconds in the CRX group vs 88 (IQR, 58-111) seconds in the CON group. CRX participants were faster in completing the TMT-B at 12 months, adjusted for baseline TMT-B and mRS score (adjusted difference in median time to completion, −3.75 [95% CI, −5.02 to −2.49] seconds).

No difference was observed in mean (SD) relative TBV change between CRX (0.13% [1.1%]) and CON (0.18% [1.1%]) groups (adjusted mean difference, −0.048% [95% CI, −0.48% to 0.38%]). During the intervention period, there were no deaths, 3 SAEs, and 1 AE of special interest (recurrent stroke or TIA) in each group. eTables 4 and 5 in Supplement 2 include details of all AEs reported during the study period collated via organ system.

No differences in exploratory outcomes at 12 months were observed. ADAS-Cog scores were lower (better) for CRX compared with CON participants, including 32 of 49 CRX participants (65.3%; median, 9.3 [IQR, 6.7-13.4]) vs 31 of 55 CON participants (56.3%; median, 10.3 [IQR, 8.5-14.5]; adjusted difference in medians, −1.00 [95% CI, −1.35 to −0.65).

## Discussion

In this randomized clinical trial, our CRX intervention was safe and feasible but did not preserve HV more than the balance and stretching control. There are many possible explanations for this neutral finding, including chosen intervention dose, intensity, duration, comparator, and study power. Our sample size was predicated on observational data,^19,30^ not interventional trials. In healthy midlife, estimated hippocampal atrophy rates are 0.98% per year.^33^ Both the CRX (−0.26%) and CON (−0.11%) groups had less hippocampal atrophy than reported during a similar 2-month timeframe in stroke survivors (−1.25%).^19^ The choice of an active balance and stretching control as the comparator—chosen to keep participants blinded to intervention arm—may have obscured any potential effects of CRX on HV. The comparable atrophy rates suggest that our active control was equivalent for brain volume preservation,^3^ consistent with increasing evidence that balance training has brain benefits.^34,35^ The sociomotivational benefits of exercise interventions may be similar to the induced cardiorespiratory benefits^36^: time spent with exercise staff was carefully matched for this study.

The CRX intervention was associated with better adjusted performance on the TMT-B and on our exploratory cognitive outcome, the ADAS-Cog, at 12 months. Both CRX and CON groups entered the study with comparable TMT-B times to completion. The mean difference of 3.75 seconds faster on adjusted median time supported a potential protective effect of the CRX intervention on processing speed and executive function. This persisting effect during the first poststroke year has not been previously demonstrated in stroke survivors. It accords with results of exercise trials in people with Alzheimer dementia,^37^ although trials in people with vascular cognitive impairment have shown variable results.^38^ Global cognitive measures improved in people with vascular cognitive impairment^39^ in a graded walking intervention but were not sustained at 6 months. A 12-week exercise intervention in people with TIA or mild stroke improved executive function but not processing speed.^40^

The dissociation of cognitive benefits from HV is not surprising. TMT-B performance is a highly sensitive measure of distributed frontoparietal network function reliant on white matter health^26^ and does not specifically entrain hippocampal processing. We found no HV effect modification in prespecified subgroup analyses, including age, sex, substudy, and educational level. Exercise therapists could interpret this as there being no evidence preventing the intervention from being delivered at home or in clinic, regardless of educational attainment, expanding options for clinicians and stroke survivors.

### Strengths and Limitations

Our study has many strengths. Our excellent retention meant that for our primary outcome, data were robust. Time spent with exercise professionals was closely matched with good treatment fidelity. Our broad inclusion criteria meant that we included people from diverse educational and social backgrounds and allowed examination of potential effects from educational attainment. Pandemic-induced protocol changes also allowed us to compare in-person vs remotely delivered interventions. Our close phenotyping of participants with sociodemographic, imaging, cardiometabolic, mood, physical activity and fitness, functional, cognitive, inflammatory and neurodegenerative biomarkers, dietary, and microbiome analyses will allow future unpacking of the effects of the intervention in post-hoc studies.

This study also has some limitations. While we had few missing data for our primary outcome, there were missing data for our secondary cognitive outcome. Our ability to administer cognitive testing was severely limited for the 2020-2022 period, as it had restrictions on in-person testing and time spent with participants due to physical distancing requirements.^28^ We were not powered to fully determine the relationships between in-person vs remotely delivered interventions. The random observed imbalance of atrial fibrillation between treatment arms could have affected training response or achieved intensity. It is difficult to interpret whether our reported TMT-B difference would represent a minimally clinically important one, as there is no documented minimally clinically important difference for this metric when adjusted for baseline performance or examined change over time.

We recruited fewer women. Obstacles to recruitment of women and gender-diverse individuals are well documented in cardiovascular^41^ and stroke trials,^42^ and sex-specific differences are understudied in cardiovascular disease. Our participants were highly educated (median, 15 [IQR, 12-17] years of education), representative of the Australian mean educational level of 13.7 years. Our MRI-dependent primary outcome and requirement to be ambulatory at 2 months excluded people with MRI contraindications and severe stroke, potentially limiting the generalizability of our findings. The proportion of men in the CON group was greater than in the CRX group, as randomization was not stratified by sex. Stroke lesion volume was greater in the CRX group.

## Conclusions

In this randomized clinical trial, we demonstrated that a CRX intervention is safe and feasible and shows promising signs of cognitive efficacy; however, there was no difference in the primary outcome, change in HV volume, between the CRX and CON groups. The study design is scalable and readily translated into phase 3 trials. More than 60 million people worldwide have dementia, expected to increase to 152.8 million by 2050.^43^ There is a complex relationship among vascular risk factors, physical inactivity, stroke, and dementia,^13^ and at least 45% of dementia risk is modifiable. The finding that a CRX intervention may lead to better cognitive outcomes at 12 months is novel and of great importance to the stroke community. Exercise therapists could aim for CRX interventions, encouraged by improved cognitive outcomes, but be reassured that balance and stretching interventions offer comparable brain volume effects. Studies in survivors of both ischemic and hemorrhagic stroke examining differences in dose, timing, and duration may provide further evidence of the brain benefits of exercise after stroke.

## Notes

Supplement 1.
Trial Protocol

Supplement 2. **eTable 1.** Per-Protocol Included Participants: Baseline Demographics, Relevant Brain Volumes, Vascular Risk Factors, and Cognitive Tests **eTable 2.** Summary of Exercise Intervention Per-Protocol Included Participants **eTable 3.** Summary of Primary, Secondary, and Exploratory Outcomes Per-Protocol Analysis **eTable 4.** Adverse Events Categorized by Organ Type During and Post Intervention **eTable 5.** Serious Adverse Events Categorized by Organ Type During and Post Intervention **eFigure 1.** Sensitivity Analysis for Change in Hippocampal Volume From 2 to 4 Months Modified Intention-to-Treat Population **eFigure 2.** Sensitivity Analysis for Change in Hippocampal Volume From 2 to 4 Months Per-Protocol Population **eFigure 3.** Forest Plots for Prespecified Subgroup Analyses for Per-Protocol Population **eFigure 4.** Illustration of Treatment Protocol and Progressive Overload **eFigure 5.** Stroke Lesion Overlap Map for Participants in the CON and CRX Groups **eAppendix 1.** MRI Scanner and (T1-Weighted MPRAGE) Acquisition Details **eAppendix 2.** MRI Original Sequences

Supplement 3.
Nonauthor Collaborators

Supplement 4.
Data Sharing Statement
