# Supplementary material for: Poststroke Cardiorespiratory Exercise for Brain Volume and Cognition: A Randomized Clinical Trial
Source: JAMA Netw Open. 2025 Aug 26;8(8):e2528907. doi: 10.1001/jamanetworkopen.2025.28907 (PMC12381666; doi:10.1001/jamanetworkopen.2025.28907)
Supplement: Supplement 1. — Trial Protocol [file jamanetwopen-e2528907-s001.pdf]

PROTOCOL

Post Ischaemic Stroke Cardiovascular Exercise Study – Zoom Delivered Intervention Against Cognitive Decline (PISCES-ZODIAC)

(The brain benefits of home-based exercise training after stroke)

Protocol Number: HREC/16/Austin/45

Version: 14

Date: 07/06/2023

Author/s:

Principal investigators: Professor Amy Brodtmann, Dr Philip Choi (Eastern Health), Professor Tissa Wijeratne (Western Health), Prof Gavin Williams (Epworth Health)

Associate investigators: Professor Julie Bernhardt, Dr Liam Johnson, Dr Emilio Werden, Prof Vincent Thijs, Dr Kathryn Hayward, Dr Naotaka Izuo, Dr Sharon Kramer, Ms Alex Billett, Dr Barbara R Cardoso, A/Prof Francine Marques, Kimberley Adkins, Ms Ruwayda Haibe, Ms Elizabeth McInerney, Ms Laura White, Ms Stephanie Tucker

Sponsor/s:

Monash University

(funding provided by The Heart Foundation and the NHMRC)

CONFIDENTIAL

This document is confidential and the property of Monash University. No part of it may be transmitted, reproduced, published, or used without prior written authorization from the institution.

Statement of Compliance

This document is a protocol for a research project. This study will be conducted in compliance with all stipulation of this protocol, the conditions of the ethics committee approval, the NHMRC National Statement on ethical Conduct in Human Research (2007) and the Note for Guidance on Good Clinical Practice (CPMP/ICH-135/95).

Table of Contents

Contents

Table of Contents 2

1. Glossary of Abbreviations & Terms 6

2. Study Sites 7

   a. Study Location/s 7

3. Introduction/Background Information 8

   a. Lay Summary 8

   b. Introduction 9

   c. Background information 9

4. Study Objectives 13

   a. Hypothesis 13

   b. Study Aims 14

   c. Outcome Measures 14

5. Study Design 15

   a. Study Type & Design & Schedule 15

   b. Standard Care and Additional to Standard Care Procedures 18

   c. Randomisation 21

   d. Study methodology 21

6. Study Population 31

   a. Recruitment Procedure 31

   b. Inclusion Criteria 32

   c. Exclusion Criteria 32

   d. Consent 32

7. Participant Safety and Withdrawal 33

   a. Risk Management and Safety 33

   b. Adverse event management 35

c. **Handling of Withdrawals** **38**

d. **Replacements** **39**

8. Statistical Methods **39**

a. **Sample Size Estimation & Justification** **39**

b. **Power Calculations** **39**

c. **Statistical Methods To Be Undertaken** **39**

d. **Qualitative Analysis** **40**

9. Storage of Blood,Tissue and Stool Samples **40**

a. Details of where samples will be stored, and the type of consent for future use of samples 40

10. Data Security & Handling **41**

a. **DATA COLLECTION** **41**

b. **Details of where records will be kept & How long will they be stored** **41**

c. **Confidentiality and Security** **41**

d. **Ancillary data** **41**

11. References **42**

STUDY SYNOPSIS
(please provide a brief information)

Title:	Post Ischaemic Stroke Cardiovascular Exercise Study – Zoom Delivered Intervention Against Cognitive Decline (PISCES-ZODIAC): The brain benefits of home-based exercise training after stroke
Short Title:	PISCES-ZODIAC
Design:	Intervention follow-up study
Study Centres:	The Florey Institute of Neuroscience and Mental Health, Melbourne Brain Centre; Sunshine Hospital, Western Health, Monash University & Baker Institute, The Alfred Centre.
Hospital:	Austin Hospital; Eastern Health Stroke Units; Sunshine Hospital; Epworth HealthCare Camberwell
Study Question:	Does home-based aerobic exercise help to slow down cognitive decline and brain volume loss after ischaemic stroke?
Study Objectives:	Establish whether a prescribed home-based aerobic exercise programme can protect against brain atrophy and cognitive decline post-ischaemic stroke.
Primary Objectives:	The benefit of exercise on post-stroke cerebral atrophy and brain function recovery. Four-month post-stroke (post-intervention) brain volume change will be a primary outcome measure (hypothesis 1). Concurrent neuropsychological testing will indicate whether the clinical neurocognitive profile aligns with measures of brain volume.
Secondary Objectives	The link between post-stroke physical activity levels, brain volume change, fitness level, sleep, diet, and incident end-organ disease (all are related to Secondary hypotheses) will be investigated through the secondary outcome measures of silent or recurrent brain infarction, cardiac diastolic function, ambulatory BP, aerobic capacity, dietary intake, gut microbiome and objective and subjective measures of sleep. Blood biomarkers reflecting endocrinological and neurological health will also be measured.<br><br>A qualitative sub-study will be undertaken to evaluate the feasibility of post-stroke home-based exercise from the patient's perspective.

Inclusion Criteria:	• Ischaemic stroke (first or recurrent);<br>• Aged over 18 years;<br>• Able to attend 3 study sessions over 10 months;<br>• Motivation and willingness to participate in the study protocol;<br>• No other prior neurological or psychiatric disease, including dementia;<br>• Able to give consent or have next-of-kin/caregiver consent by proxy.<br>• Able to nominate somebody as an emergency contact
Exclusion Criteria:	• Medical co-morbidities precluding participation in exercise intervention (e.g., severe cardiovascular disease), or making survival for 4 months post-stroke unlikely;<br>• Regular exclusion criteria for MRI (e.g., implanted metal, severe claustrophobia);<br>• mRS >3.
Number of Planned Subjects:	120
Investigational product:	An understanding of the benefits from prescribing a home-based physical activity regimen to specific post-stroke patient sub-types. Furthermore, an ability to predict the areas of physical and neuropsychological function that can be assisted by exercise treatment.
Safety considerations:	Severe medical comorbidities (e.g., cardiovascular disease); Regular exclusion criteria for MRI. Participants will be observed throughout the study for potential Adverse Events (AEs) and Serious Adverse Events (SAEs) and these events will be recorded.
Statistical Methods:	ANCOVA (with exercise intervention 'Physical fitness training' vs. 'Balance training' as a factor), controlling for baseline status, will be used to measure brain volume changes from baseline to 4 months post-stroke (hypothesis 1). Regression analyses will determine the association between brain volume, brain function, and end-organ disease measures with treatment group as the independent variable (secondary hypotheses). Further regression modelling will explore individual contributing effects of exercise on end-organ measures, aerobic capacity, sleep, diet, blood biomarker levels, and gut microbiome composition.

Subgroups:	Based on exercise intervention: approx. 60 receive 'Physical fitness training', approx. 60 receive 'Balance training'.<br><br>All participants will be invited to partake in the qualitative sub-study into the feasibility of post-stroke home-based exercise intervention.

1. GLOSSARY OF ABBREVIATIONS & TERMS

Abbreviation	Description (using lay language)
Aerobic exercise	Physical activity, ranging from low-high intensity levels, that requires the body to efficiently pump oxygenated blood to working muscles. This is opposed to 'Anaerobic exercise', which occurs in the absence of blood-oxygen supply.
Baseline Assessment	Approximately 2 months post-stroke is the time point of the Baseline Assessment (i.e., initial assessment) of participants in the study and the time point at which the exercise intervention is delivered. Throughout this Protocol, the terms '2 month post-stroke' and 'Baseline' will be used interchangeably.
BDNF	Brain Derived Neurotrophic Factor – Protein found in the central and peripheral nervous systems that supports neurons, encourages neural growth, and enhances neural connections.
BP	Blood Pressure – pressure applied to blood vessel walls by circulating blood.
Hb1A	Glycated Haemoglobin – marker of 3-month average plasma glucose concentration
LV	Left ventricle – Large pumping chamber on the left side of the heart
LVH	Left ventricular hypertrophy - enlargement and thickening of the left side of the heart.
mRS	Modified Rankin Scale score – post-stroke degree of disability and/or dependence in daily activity
MRI	Magnetic Resonance Imaging – a brain scan that uses changes in magnetic fields to measure brain volume and thickness.

2. STUDY SITES

a. STUDY LOCATION/S

Site	Address	Contact Person	Phone	Email
Monash University, Central Clinical School	The Alfred Centre, Room 318, Level 5<br>99 Commercial Road, Melbourne<br>VIC 3004	Prof Amy Brodtmann	03 9903 9330	amy.brodtmann@monash.edu
		A/Prof Francine Marques	03 9905 6958	francine.marques@monash.edu
		Dr Barbara R Cardoso	03 9902 4264	barbara.cardoso@monash.edu
		Ms Kimbderly Adkins	0419 148 085	kim.adkins@monas.edu
		Ms Laura White	0426 094 766	laura.white@monash.edu
		Ms Stephanie Tucker	0401 727 914	stephanie.tucker@monash.edu
		Ms Ruwayda Haibe	NA	ruwayda.haibe@monash.edu
		Ms Elizabeth McInerney	03 9903 0923	elizabeth.mcInerney@monash.edu
The Florey Institute of Neuroscience and Mental Health, Melbourne Brain Centre, Austin Campus	245 Burgundy Street, Heidelberg VIC 3084	Dr Emilio Werden	03 9035 7275	werdene@unimelb.edu.au
		Prof Julie Bernhardt	03 9035 7072	j.bernhardt@unimelb.edu.au

		Dr Liam Johnson	03 9035 7390	liam.johnson@florey.edu.au
Austin Health	Austin Hospital, 145 Studley Rd, Heidelberg VIC 3084	Prof Vincent Thijs	03 9496 4824	vincent.thijs@florey.edu.au
Eastern Health	Box Hill Hospital, 8 Arnold Street, Box Hill VIC 3128	Dr Philip Choi	03 9895 3352	philip.choi@easternhealth.org.au
Western Health	Sunshine Hospital 176 Furlong Road, St Albans VIC 3021	Prof Tissa Wijeratne	0430 048 730	twi@unimelb.edu.au
Epworth Health	Epworth Camberwell 89 Bridge Rd Richmond VIC 3121	Prof Gavin Williams	03 9426 6094	gavin.williams@epworth.org

3. INTRODUCTION/BACKGROUND INFORMATION

a. LAY SUMMARY

The research shows that after a stroke, there can be a continual reduction in brain volume (this is called ‘atrophy’) and as a result ongoing decline in thinking. A decline in thinking or ‘cognitive function’ can, in severe cases, progress to a cognitive dementia. The major risk factors for post-stroke brain atrophy and cognitive decline are more strokes, high blood pressure (hypertension), and low rates of physical activity. In this project, we will examine if exercise after stroke can modify risk factors that may lead to post-stroke brain atrophy.

We will recruit 120 participants who have suffered ischaemic stroke within the past 2 months. At the 2 month post-stroke time-point, participants will be randomly allocated to one of two respective exercise groups, named according to the nature of the exercise programme: ‘Physical fitness training’ vs. ‘Balance training’. The exercise programme will follow an 8-week course. At two subsequent assessment visits over the next 10 months, brain volume, cognitive function, cardiovascular well-being, sleep, diet, the gut microbiome, and general body fitness will be measured.

This means that over 1 year post-stroke, we will be able to compare whether aerobic exercise (that is exercise requiring the efficient delivery of blood-oxygen to working muscles), has a positive impact on preserving brain volume and function, as well as general physical well-being. We will use the latest brain imaging techniques to accurately measure brain volume and thickness. Cognitive testing will be used to gauge brain function. Blood and stool sampling allow us to check whether there are any changes in key brain-and-body bio-chemicals. Sleep will be assessed to determine its moderating role in stroke rehabilitation. Fitness capacity will be assessed pre- and post-exercise intervention. Study investigators have both research and clinical experience in the fields of stroke and exercise to ensure both the scientific rigor and participant safety.

This research will add to our understanding of two of the major causes of death, disability and reduced quality of life in our society: dementia and stroke. Furthermore, the feasibility of post-stroke home-based exercise from the participant's perspective will be evaluated. One of the most important things about this research is its novelty – unanswered questions will be tackled.

b. INTRODUCTION

The evidence is compelling: vascular burden is the greatest determinant of late life cognition. The imaging hallmark of neurodegeneration is brain atrophy. Cerebrovascular disease is associated with accelerated rates of structural brain aging, such as atrophy and white matter ischaemic burden. We have demonstrated increased rates of total and regional brain atrophy following ischaemic stroke [1-3]. Patients lose brain volume at a greater rate than control participants, and this volume loss is correlated with cognitive deficits. But not everyone atrophies at the same rate, and not everyone dements. The major risk factors that have emerged for post-stroke brain atrophy and cognitive decline are recurrent stroke, hypertension, and low rates of physical activity.

In this project, we will examine these modifiable risk factors that may predict post-stroke brain atrophy. Using brain volume and brain connectivity measures at 4 months and 12 months after stroke, we will examine the effects of a targeted cardiovascular home-based exercise intervention on brain volume. We will chart cumulative vascular burden via advanced brain imaging, ambulatory blood pressure (BP) monitoring, and interrogate the links between activity levels, hypertension, cardiac diastolic dysfunction, cognition, aerobic capacity (i.e., fitness), diet, gut microbiome, and key physiological biomarkers.

The original PISES Study demonstrated good retention and attendance rates, but we found two major factors have hindered recruitment. Over one third of people approached to participate listed 'inconvenient program location' or 'time commitment demanding' as reasons for not participating. The impact of COVID-19 has further limited uptake in a centre-based intervention. In response, PISCES-ZODIAC has been designed to overcome these barriers by delivering the intervention via one-on-one telehealth consult in the convenience of the participant's own home by a trained exercise professional.

c. BACKGROUND INFORMATION

Cumulative vascular brain burden, cognition and brain volume

Each of the major cardiovascular risk factors has now been independently associated with cognitive impairment in later life. Mid-life hypertension is a leading cause of cognitive impairment [4], with deficits detectable after only a few years of raised blood pressure [5]. Hypertension causes small vessel disease, which in turn is associated with gradual, progressive cognitive impairment, functional decline and brain atrophy [4]. Hypertension causes progression of small vessel disease over 3 years, and this is associated with cognitive decline. Higher evening diastolic BP on ambulatory monitoring is associated with the greatest risk [5]. Rapid accumulation of white matter disease and cognitive decline are linked to ambulatory BP and physical activity over 4 years [6]. This finding prompted a study of the use of ambulatory monitoring – rather than clinic tests – to reduce cerebrovascular disease [7]. Yet most stroke patients do not have ambulatory blood pressure monitoring following discharge.

Emerging themes from the Cognition and Neocortical Volume After Stroke (CANVAS) study

Researchers from the Alzheimer's Disease Neuroimaging Initiative (ADNI) study found that neurodegeneration, as measured by atrophy on MRI, both *precedes and parallels cognitive decline*, and concluded that serial MRI is the most useful modality for longitudinal dementia studies [8-9]. Brain volume loss after stroke has been controversial. It has only been with the advent of longitudinal studies that this progressive neurodegeneration has been documented *in-vivo*. The Melbourne-based Cognition and Neocortical Volume after Stroke (CANVAS) study, led by Prof Brodtmann [3], has been critical in describing these changes following stroke. The CANVAS study is a longitudinal study correlating cognitive performance and brain volume changes after ischaemic stroke [1, 3]. One hundred and thirty five participants with ischaemic stroke and 40 age- and sex-matched healthy control participants are tested on 4 occasions over 3 years with identical cognitive tests and advanced MR imaging. The primary hypothesis posits that change in regional and global brain volume between the 3 months and 3 year testing time-points correlates with cognitive performance.

CANVAS demonstrates advantages of using brain volume as an outcome measure

Brain volume is an excellent proxy measure of subsequent cognitive outcome [8-9]. In the ADNI and Australian Imaging, Biomarker & Lifestyle Study of Ageing (AIBL) studies, declining total and hippocampal volume are strongly associated with the development of cognitive impairment [8-10]. Brain volume loss precedes and predicts cognitive decline [8]. This is being reflected in the CANVAS dataset, where we are seeing hippocampal volume loss at 3 months reflected in memory measures at both at 3 months and at one year.

Rapid brain volume loss after stroke

Even within the first 3 months, we have found that brain volume changes are occurring at a much faster rate than controls [1]. Significant hippocampal and thalamic volume loss occurs in this early period, throwing further doubt on the concept that brain volume at 3-6 months after stroke can really represent a true baseline. We have found that there are progressive changes in brain volume after stroke, and this persists 1 year following the event [1]. Patients lose brain volume at greater rates than controls [1]. The hippocampi and thalami appear uniquely vulnerable, as well as prefrontal cortical regions. Hippocampal volumes can decline 5-10% over 1 year. This volume loss is associated with impairment on memory testing. Some patients lose hippocampal volume between baseline and 3 months after stroke, only to recover at 1 year post-stroke. Why do some patients continue to lose brain

volume, and others don’t? What aspects of their risk factor profile are modifiable, and what can we treat to prevent this atrophy that precedes and predicts cognitive decline?

Physical activity, cognition and brain volume

Physical activity consistently emerges as a key modifiable factor for brain health. Regular, lifelong aerobic exercise reduces risk of all-cause dementia [11]. We have found an association between physical activity measures and cognition at 3 months following stroke [12]. We found that the percentage of the day spent active was correlated with both memory and executive function (Stroke Society Association meeting, September 2014). Most interestingly, we have also found that physical activity measures are associated with changes to the resting state functional MRI (rsfMRI) derived functional connectivity of the dorsal attentional network – see Figure 1. More time spent active was associated with increased connectivity in the left inferior parietal region, near the intraparietal sulcus: a critical node in the dorsal attention network [45]. Physical activity, especially aerobic exercise, has many benefits, making it difficult to unpack which aspects are most efficacious in preventing brain atrophy and cognitive decline. Activity improves blood pressure control, and can avert conversion from pre-hypertension to hypertension; it reduces inflammatory markers; it increases socialization thereby both increasing social stimulation and reducing the incidence of depression; it improves cardiovascular capacity, improving vascular reactivity, causing cardiac remodeling and increasing left ventricular (LV) diastolic relaxation; it improves insulin resistance, reduces adiposity, increases lean muscle mass and normalises lipid profiles, reducing LDL and increasing HDL cholesterol.

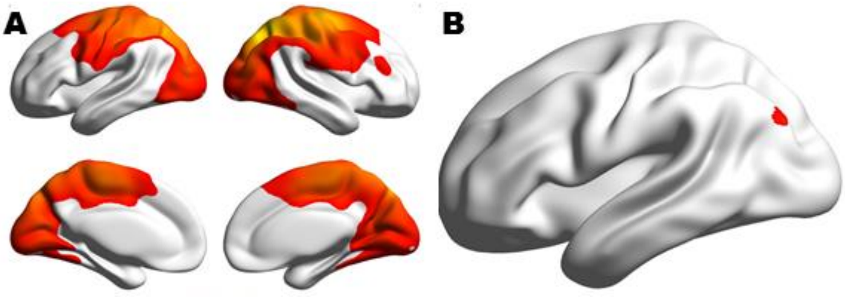

Figure 1 Physical activity modulates attention network: A: rsfMRI derived dorsal attention network (DAN) in 64 stroke patients at 3 months (height uncorrected p<0.001, FDR cluster corrected p<0.05). **B:** Significant modulation with physical activity levels in right inferior parietal lobe adjacent to IPS, a critical node in the DAN.

Blood biomarkers: BDNF and HbA1c

Biomarkers, are biological markers of pathophysiological change. Two biomarkers of interest in the realm of stroke and neurophysiological recovery are Brain-Derived Neurotrophic Factor (BDNF) and Glycated Haemoglobin (HbA1c).

<u>BDNF</u>: BDNF is a growth factor involved in the development and survival of neurons and neuronal pathways. BDNF exists within the central nervous system and in peripheral circulation. Majority of stroke-related BDNF studies have primarily been undertaken in animal models, revealing a stroke-associated increase in brain BDNF levels which may also be evident in the circulation [13]. In humans, there is evidence that blood serum BDNF correlates with a reduction in cardiovascular risk 90 days post-stroke [14]. In patients with

coronary artery disease, higher serum BDNF levels have been found in patients with better cardiopulmonary capacity and was related to stronger cognitive function [15]. Whether neurological recovery accompanies these improvements is not clear or not well-supported [14]. No study has yet investigated the role BDNF might have in post-stroke recovery using advanced neuroimaging in combination with cognitive assessment, and a targeted exercise intervention.

HbA1c: HbA1c reflects 3-month average plasma glucose concentration and is commonly a marker of glycaemic control. In patients with Type 2 Diabetes Mellitus (T2DM), aerobic exercise can reduce HbA1c levels, thereby suggesting improved management of glycaemic levels [16]. Heightened HbA1c concentrations $\geq$7% appear to show a threshold risk for stroke in people without a history of diabetes [17]. We are currently conducting a study, based at Austin Health, investigating the neurological and neuropsychological outcome of cardiovascular disease in diabetes. There is a strong rationale to explore plasma glucose control in patients who have stroke and the potential benefits of exercise intervention.

Both BDNF and HbA1c can be sampled via blood collection, which is often already being undertaken as a normal part of a study protocol with minimal impact or discomfort towards participants.

Genetic and vascular risk factors

Apolipoprotein E (ApoE) is a glycoprotein responsible for lipid transport in the brain and other organs. It exists in three isoforms (E2, E3 and E4) encoded by three alleles (ε2, ε3, and ε4) on chromosome 19. It confers considerable difference in terms of function and associated risk of disease. The ε4 allele was found to be a significant genetic risk factor in the development of sporadic Alzheimer’s Disease (AD) [18]. A single ε4 allele increases the risk of AD approximately 3 times while two alleles confer up to 12 times the risk compared to non-carriers. It also increases the risk of vascular disease. As a result of impaired cholesterol transport and metabolism, both APOE ε2 and APOE ε4 accelerate atherogenesis. There is now evidence that APOE genotype is related to progression of chronic ischaemic white matter lesion load, as well as being associated with increased hippocampal atrophy, and cognitive decline [19, 20, 21].

ApoE will be sampled via blood, once-off at the first assessment visit (2 months post-stroke), as part of the one blood collection procedure. If this cannot be done, for any reason, we will try again at the second (4 months post-stroke) or third (12 months post-stroke) visits.

Sleep

Poor sleep symptoms are both a risk factor and consequence of stroke. Chronic sleep disturbance is associated with inflammatory conditions such as cardiovascular disease [22], and both sleep disordered breathing and snoring are risk factors for stroke in particular [23, 24]. Poor sleep quality is also associated with increased risk of Alzheimer’s Disease, cognitive decline, and increased cortical atrophy in community dwelling adults [25, 26]. In first-incident stroke patients, research shows poor subjective sleep symptoms are reported in over 70% of patients, and those with poorer sleep evidenced greater functional impairment across all strength and coordination tests assessed [27]. Further, research shows that poor sleep quality is considered one of the top five risk factors correlated with poor prognosis for ischemic stroke patients between 18-45 years [28]. As such, sleep may be an important

component to stroke rehabilitation, and there is strong rationale to measure sleep changes as a moderator or mediator of improvement in brain health.

Diet

Associations between diet and cognitive decline are strongly suggested in numerous epidemiological studies and interventional trials. In fact, diet is one of the most relevant modifiable risk factors for early cognitive changes in non-demented older people [29]. It has been projected that controlling seven of the most relevant modifiable risk factors, including mid-life obesity, physical inactivity, smoking, low educational attainment, depression, diabetes mellitus, and hypertension, would delay the onset of dementia and reduce its incidence by up to 30% [**30**]. The consumption of a healthy diet, characterised by high intake of nutrient-dense foods such as fruits, vegetables, legumes and cereals, and low intake of high fat and high sugar foods, has been associated with higher brain volume in older adults [31]. Given the strong evidence demonstrating the importance of diet for brain health, it is imperative to assess diet as a mediator of stroke rehabilitation.

Gut microbiome

An emerging field of significant research interest involves investigation into the function and dysfunction of the gut microbiome, and its associations with brain health [32]. In line with current theories of the gut-brain axis, gut microbiome health has been linked to onset and progression of neurological disorders, and particularly neurodegenerative diseases such as Alzheimer's, Parkinson's, amyotrophic lateral sclerosis, and Huntington's disease [32]. The associations between the gut microbiome and cognition and outcomes following stroke have received less attention.

Previous research calls for treating the gut as the missing link between exercise and neurodegenerative disorders, particularly because it is a changeable ecosystem and can be targeted through a low-cost therapy such as an exercise program. We hypothesise that alterations in gut microbiota (bacterial markers of gut health) will be associated with the rate and extent of post-stroke cognitive decline. Given the links between exercise, diet, stress and gut health, this relationship is also expected to be moderated by (a) PISCES exercise intervention group or physical activity level as measured by subjective physical activity questionnaires and objective physical activity data from 7-day actigraph monitor wearing; (b) dietary composition as measured with a dietary monitoring app over time period; and (c) psychological factors including mood and self-reported quality of life.

Emerging themes for current study: aims, primary and secondary outcome measures

Recurrent stroke; poorly controlled hypertension; low amounts of physical activity, poor sleep: each of these is independently associated with both cognitive decline and brain atrophy. In addition, each of these represent a modifiable risk factor, one that if demonstrated to contribute to post-stroke regional and total brain volume loss could be readily identified and treated.

4. STUDY OBJECTIVES

a. HYPOTHESIS

The hypotheses of this research project are that:

Primary hypothesis

1. Participants who receive the Physical fitness training intervention will have preserved regional and global brain volume at 4 months post-stroke compared to Participants in the Balance training group.

Secondary hypothesis

i. Brain volume will be correlated with BP and incident brain infarction at 4 months and 1 year post-stroke;

ii. Incident end-organ disease (silent or recurrent brain infarction, ambulatory blood pressure (BP), and cardiac structural disease) at 4 months and 1 year post-stroke will be reduced by aerobic exercise;

iii. Incident physical activity levels, normotension, normal cardiac indices, higher aerobic capacity, and better sleep profiles will be associated with preserved brain volume at 4 months and 1 year post-stroke.

b. Study Aims

Using brain volume and brain connectivity measures at one year after stroke, the aims of this research project are:

1. Examine the effects of home-based aerobic exercise intervention delivered at 2 months post-stroke on brain volume 4 months after stroke, and the stability of such effects at 1 year after stroke;
2. Document vascular burden using 3 end-organ effects: incident brain infarction, cardiac diastolic dysfunction, and ambulatory BP;
3. Examine the link between incident physical activity, aerobic fitness, sleep, and brain volume;
4. Examine the effects of exercise on blood pressure and cardiac diastolic function 4 months and 1 year after stroke;
5. Measure the change in BDNF and HbA1c levels 4 months and 1 year post-stroke and if exercise affects these;
6. Examine the association between physical activity, dietary intake, gut microbiome and brain volume.

An additional aim, which has been framed as a sub-study nested within the greater protocol, will enable us to capture any specific factors that influence participation in, or adherence to, post-stroke home-based exercise regimens.

c. Outcome Measures

Our primary outcome measure is:

- Brain volume at 4 months post-stroke.

Our secondary outcome measures are several end-organ disease measures:
- Silent or recurrent brain infarction;
- Cardiac diastolic function;
- Ambulatory BP;
- Blood BDNF and HbA1c;
- Sleep duration, variability, efficiency, and onset latency;
- Dietary intake and gut microbiome

Qualitative:
- Understand the feasibility of prescribing post-stroke home-based exercise.

5. STUDY DESIGN

a. STUDY TYPE & DESIGN & SCHEDULE

This is a randomised controlled, multi-site longitudinal study of 120 ischaemic stroke participants (aged >18 years) followed for 1 year (12 months) post-stroke, who at 2 months post-stroke receive an 8-week intervention programme of either Physical Fitness training (approx. 60 participants) or Balance training only (approx. 60 participants). The 2 home-based exercise programmes denote the 2 experimental groups into which participants will be randomly assigned. Participant recruitment will take place at the Austin Health, Eastern Health Western Health and, Epworth HealthCare Stroke Units. MRI Brain scanning, cognitive testing, and assessment of aerobic capacity, as well as blood sampling (to determine HbA1c level, BDNF level, APOE status and genetic risk assessment) will be performed at the Florey, Melbourne Brain Centre, Austin Campus; Sunshine Hospital, Western Health, Monash University & Baker Institute, The Alfred Centre. Some assessments (e.g., demographic interview and cognitive test battery) may be conducted at participants' homes to minimise their burden. Participants will be provided with devices to record their activity levels (for 7 days) and ambulatory BP (24 hours) at times around the scheduled study visits. To minimise inconvenience to participants, arrangements will be made to fit the ambulatory BP device on the first part of each study visit so that it can be returned at the second part of the study visit.

Participants will be given a stool collection kit to take home at each of the 2-, 4- and 12-month time-points. Participants will then return the sample to the study site closest to their home (i.e. Florey, Melbourne Brain Centre, Austin Campus; Sunshine Hospital, Western Health, or Monash University, The Alfred Centre) when returning the BP (24 hour) device.

Participants who have a smartphone will be shown how to download the app (Research Food Diary, Xyris Software, Australia) used for dietary assessment. They will also receive printed instructions about the app's basic function. They will be asked to complete a 3-day food diary (2-week days and 1 weekend) in the week following their 2-month, 4-month and 12-month time points, after they have collected a stool sample.

Timelines and feasibility

The 8 week intervention will commence at 2 months after stroke (see Figure 1. Experimental design and participant timeline). At the 2 month, 4 month, and 1 year post-stroke time-points,

we will capture brain images with MRI, perform ambulatory BP measurements and cognitive testing, assess aerobic capacity, fit Actiwatches, deliver the 7-day Sleep Diary and sleep questionnaire, and explain how to use the Research Food Diary app (sleep and dietary assessments to be completed at home). Blood samples will be collected at each time-point to check for concurrent changes in levels of BDNF and HbA1c. Additional blood (10ml) will be collected at the first (2 months post-stroke), second (4 months post-stroke), *or* third (12 months post-stroke) to test for APOE status and genetic risk. Stool samples will be collected by participants at home after each time point and they will be returned by the participant to the study site with the 24-hour BP device.

Figure 1. Experimental design and participant timeline

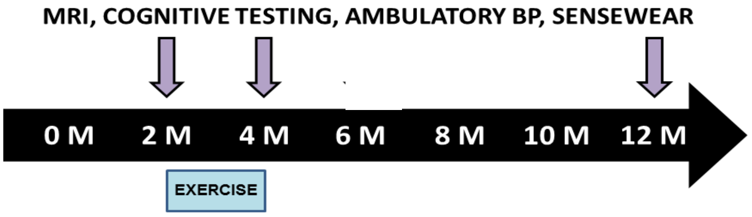

Note: Participants will be recruited after stroke (0 M=months) and randomised to an 8 week physical activity intervention, commencing at 2 M

Participant home visits

One home visit will take place 1-week prior to the initial baseline assessment and prescription of exercise programme at the 2 month post-stroke timepoint. At the home visit, an Exercise Professional (EP) and/or Occupational Therapist (OT) will assess the safety of the area the participant will be exercising in and minimise potential risks.

Home Visit Risk Management Procedures and Guidelines will be followed as per The Florey's Occupational Health and Safety Manual Section 1 (please refer to https://unimelbcloud.sharepoint.com/sites/florey/ohs/).

A friend or family member will attend the initial home visit. The EP/OT will determine the level of involvement of this contact, depending on the participant's level of independent function and mobility. The emergency contact will either a) solely provide their contact details in case of emergency or b) act as a study partner and will need to be in the participant's home for each of the sessions. Study partners will sign a separate consent form.

Recruitment

Our experience from the CANVAS and other imaging stroke studies suggest that around 30-40 stroke participants can be recruited into an intensive imaging study over a 1 year period.

A pre-study assessment questionnaire will be completed to determine eligibility in the study. This assessment addresses very broad aspects of MRI eligibility (e.g. pacemaker or severe claustrophobia). Informed consent will then be obtained from participants before any

participation in the study protocol. A thorough MRI-safety screening questionnaire will then be used. This will involve the collection of demographic information (e.g., name, address, contact phone number), medical information (e.g., previous medical operations and procedures, inserted devices, cardiovascular function, respiratory complications, current medications) and psychiatric information (e.g., previous diagnoses). This information will be collected to ensure that participants have no contraindications for MRI and are suitable for the study.

Challenges of high-field research MRI

Despite a stroke pool of over 1400 annual admissions between the 4 recruiting hospitals (Austin Hospital, Box Hill Hospital, Sunshine Hospital, Epworth Camberwell), many patients are excluded due to ill health, MRI contraindications, and competing trials. Whilst old surgical procedures are an accepted low risk for clinical MRI, high-field research MRI needs to be zero risk.

Blinding: By the nature of the study design, the investigators are blind to the outcome of interest. Irrespective of this, participants will be assigned a unique identifier at recruitment, and all data input and analysis will be performed blinded to participant status. MRI image analysis will be performed blinded to status. This will be unblinded when the cohort is complete and statistical analysis is performed.

Dr Liam Johnson (senior research exercise physiologist) and the trained exercise professionals, who undertake the exercise sessions with participants, will not be involved with cognitive testing, MRI analysis, or cardiovascular tests as they are not blinded to participant status.

Data storage: Hard copy identifiable data will be stored in a locked office (Prof Brodtmann's office) in a locked building at the Alfred Centre, Monash University, or with respect to Western Health participants, in an unmarked locked filing cabinet on the fourth floor of the Australian Institute for Musculoskeletal Science (AIMSS) facility. Electronic identifiable data will be stored in access-restricted data servers housed in a locked building at the Alfred Centre, Monash University, or with respect to Western Health participants, on Western Health servers. Non-identifiable electronic data (including all assessment results and biomarker results) will be saved on access-restricted data servers housed in a locked building at the Alfred Centre, Monash University, or in the case of dietary intake data, in the Department of Nutrition, Dietetics and Food at Monash University. Demographic information will be stored separately to any data that participants provide.

Attainment of study objectives

Clinical research expertise has informed the study design. Familiarity with Austin Health Eastern Health, Western Health, Epworth Camberwell, and The Alfred Centre sites will assist participant recruitment and then data collection according to HREC guidelines. The facilities at the Florey Neuroscience Institute,Sunshine Hospital, and The Alfred Centre will allow the investigators to use the latest brain imaging techniques to accurately measure brain volume and structure in post-stroke participants, with longitudinal testing allowing measurement of change over time after the prescription of an exercise intervention. Concurrent neuropsychological testing will determine whether the clinical profile of dementia and cognitive impairment aligns with measures of brain volume.

It is possible that in time, this study might be of interest to students undertaking projects as a part of Honours or PhD studies. Should any student involvement arise then HREC committees will be notified.

b. Standard Care and Additional to Standard Care Procedures

Standard Care Procedures		
**Procedure**	**Time/Visit**	**Dosage/ Volume**
MRI	Clinical protocol: Stroke Units at Austin Health, Eastern Health, Western Health, Epworth Camberwell, and Baker institute, The Alfred Centre campus	As per respective inpatient clinical care needs
Blood collection	Clinical protocol: Stroke Units at Austin Health, Eastern Health, Western Health, Epworth Camberwell, and Monash University:The Alfred Centre campus	As per respective inpatient clinical care needs

Additional To Standard Care		
**Procedure**	**Time/Visit** (post-stroke)	**Dosage/ Volume**
Ambulatory blood pressure and ECG	Baseline; 4 months; 1 year	1 per visit
MRI	Baseline; 4 months; 1 year	1 per visit
Blood for APOE genotyping, BDNF, HbA1c, future genetic and blood marker analyses	Baseline (additional 10ml sample taken for genetic testing); 4 months; 1 year	27ml for 2 month,4 month and 1 year visit Additional 10 ml (total=37ml) for 2 month, 4 month, *or* 1 year visit
Cognitive and Mood Testing	Baseline; 4 months; 1 year	1 set of tests per visit
Home-based exercise intervention (both groups)	Intervention starts at baseline and continues 3 times per week for 8	60 minutes per session

	weeks	
Aerobic capacity test (VO_2 peak graded stress test)	Baseline; 4 months; 1 year	30-60 minutes total, dependent on individual capacity
Physical activity monitoring	1 week after each testing time-point	Actiwatch monitors worn for a duration of 1 week
Sleep monitoring	1 week after each testing time-point, as well as prior to Baseline	7-day Sleep Diary and sleep questionnaire (completed at home)
Dietary intake assessment	1 week after each testing time-point	3-day food diary recorded with Research Food Diary app (completed at home)
Gut microbiome	Baseline; 4 months; 1 year	1 sample (completed at home)

Exercise groups and programmes

For ease of intervention and to increase accessibility and recruitment, the exercise intervention will be offered as a telehealth model. Both groups will undertake an 8-week exercise programme. Participants will partake in 1 hour-long exercise sessions, three-times per week, for 8 weeks. These sessions will be run by a trained exercise professional (e.g., Exercise Physiologist, Physiotherapist, or Research Assistant trained in exercise prescription). Training will be provided by Dr Liam Johnson (Associate Investigator and Senior Research Exercise Physiologist). The exercise programme will be conducted in the individuals' homes, whilst the exercise professional monitors and facilitates the session via Zoom or other videoconferencing programs. When possible, the participants' exercise preferences will be considered when designing their individual exercise program.

To ensure optimal levels of safety are achieved whilst completing home based interventions, a home visit will be completed prior to commencing the intervention. This home visit will be conducted by the exercise professional, in collaboration with Occupational Therapists and clinical specialists with extensive safety assessment experience, if required. The visit will be used to assess the safety of the area the participant will be exercising in and minimise any potential risks. Additionally, the home visit will determine if the participant requires a 'study partner' to be at home with them throughout the duration of the session, should they need help.

An exercise intervention 'pack' will be delivered to participants after the first time-point assessment. These packs will consist of everything required to complete the exercise intervention as usual, such as monitoring (HR monitors), safety assessments (blood pressure) and exercise equipment (i.e. bikes, cones, step, stopwatch). After the participant has finished the 8 week intervention, all equipment will be thoroughly cleaned prior to being

utilised again. Exercise equipment will be stored in secure locations within the Melbourne Brain Centre, AIMSS facility, and The Alfred Centre between participant use.

To ensure blinding is maintained, only Exercise Professionals will liaise with the participants regarding all organisation of home visits and equipment deliveries. Larger equipment required to complete the intervention such as bikes, will be professionally delivered to ensure OHS protocols are followed.

The exercise programmes will be individually-tailored and adjusted based on their physiological (heart rate) and affective (rating of perceived exertion) response. The initial prescription of aerobic exercise will be determined by the VO_2 peak graded exercise stress test (GXT) (See Study Methodology – Point IX). In addition, pre-study screening will identify any post-stroke physical sequelae that need to be considered in developing the exercise programme. The Balance training programme is designed to ensure all participants undertake the same duration of exercise and the same duration of contact with the health care professionals as the Physical fitness training group. Participants in both the Physical Fitness and Balance training groups will be assessed via the Physical Activity Scale for the Elderly (PASE), a validated and widely used questionnaire to track physical activity levels in an older population over a one-week period. We will administer the PASE via Zoom at the start of each week of the intervention. This will be administered to gauge activity levels outside of the intervention throughout the course of the intervention.

**Balance training group**		
Duration	Task	Example activity
5 mins	Warm-up	- Simple whole body exercises (Marching, arm swings)<br>- Gentle stretching muscle stretch, trunk side stretch
30 mins	Balance	Single-leg stand, Heel-to-toe walk
20 mins	Stretching	Active and dynamic upper and lower limb stretching
5 mins	Cool-down	Replication of some stretching done in warm-up

**Physical fitness training group**		
Duration	Task	Example activity
5 mins	Warm-up	- Simple whole body exercises (Marching, arm swings)<br>- Gentle stretching muscle stretch, trunk side stretch
10 mins	Strength: Functional exercises	Sit to stand, Incline wall push-ups
30 mins	Aerobic activity	Cycle ergometry
10 mins	Strength: Functional exercises	Sit to stand, Incline wall push-ups
5 mins	Cool-down	Replication of some stretching done in warm-up

For participants assigned to the Balance training programme, there will be no harm to the health and well-being or detriment towards post-stroke recovery as a result of not partaking in prescribed physical fitness activities. Participants are still encouraged to undertake their usual routine leisure activities; any such activity undertaken will be captured in daily activity

diaries. Furthermore, the benefits of undertaking a Balance training programme alone is more than would have been obtained if they were not involved in this study.

c. Randomisation

After the first time-point assessments, participants will be randomly allocated into one of two exercise groups using a computer generated schedule with permuted blocks of various sizes. This will be part of the electronic case report form (ECRF) hosted at a secure server at the Florey and compliant with Good Clinical Practice (GCP) guidelines for clinical trials. The randomisation will be stratified by baseline function (modified Rankin Scale score, grouped into mRS 0-1, 2-3) and baseline total brain volume (low, high).

Participants will be aware of their group allocation based on the exercise programme they are administered. Investigators who are blind to participant group will conduct cognitive testing, MRI analysis, and cardiovascular testing.

d. Study methodology

I. Pre-study screening

Stroke Unit ward lists and consultation with treating medical doctors, and Allied Health will identify inpatients who may be suitable for the study. Pre-study screening will then identify eligible potential participants according to inclusion/exclusion criteria.

A short questionnaire, comprising two parts (detailed below in Point I.a. and I.b.) will be used to ask questions related to study eligibility. This will distinguish potential participants who may be unsuitable for further MRI testing or the exercise intervention. Those potential participants that are identified as unsuitable by the study team will be informed at this stage and it will be explained that no further participation is required.

I.a. MRI safety check questionnaire

Participants will also be asked to partake in a short MRI safety questionnaire with the investigator. The questionnaire is used to obtain specific information about a participant's history of surgical procedures (e.g., name, date, location of procedure, whether implants were used). This questionnaire is crucial in determining whether participants have implants that might not be suitable for a 3T MRI scan. The investigators will submit an MRI safety check application to the radiographers at The Florey, Melbourne Brain Centre,Sunshine Hospital, Western Health, or Baker Institute, The Alfred Centre. If the radiographers determine that the participant cannot have a research 3T MRI scan (e.g., because they have an implant that has not been tested in a 3T MRI scanner), the participant will be notified and it will be explained that no further participation is required.

I.b. Demographic and medical history questionnaire

The research team has developed the study questionnaire. It will be used to obtain only the most pertinent demographic, medical, and psychiatric information required for this study. The questionnaire will be cross-checked for accuracy by medical record review if the participants have attended Austin Health, Eastern Health, Western Health, or Epworth Camberwell. The questionnaire will include questions on general health, cardiac risk, history of cognitive decline, hypertension, presence of dyslipidaemia, cardiac disease (MI, angina, HF, previous angioplasty and/or bypass surgery), and other macro-vascular complications (previous stroke or transient ischaemic attack, peripheral vascular disease). Age, sex, diabetic status,

and smoking history, drug therapy (antihypertensives, renin angiotensin system (RAS) inhibitors, statins) as well as family history of cardiovascular disease will be recorded.

In addition to the demographic questionnaire described above, study investigators will obtain information about participants' musical background (e.g., what instruments they have ever received training on, and for how long) using The Survey of Musical Experience [46]. An increasing amount of evidence now suggests that music training might be protective for the brain. For example, musicians have been found to perform better on tests of verbal and working memory, and executive functions [47-49], and music training has been associated with both structural and functional changes in multiple regions of the brain [50,51]. This may represent a possible confound for the current study, in determining the differences and changes in brain volume between and within the two exercise training groups that might be attributable to the exercise programmes versus pre-existing potential neuroprotective factors. While the impact of music expertise on cognition has been investigated in single case studies of individuals with stroke, to our knowledge, there currently exist no group studies examining this link, nor studies investigating the association between prior music training and brain volume in stroke.

II. Participant Information and Consent Form, Informed Consent

Inpatients: Whenever possible, participants will be approached to take part in the study while they are inpatients at Austin Health, Eastern Health, Western Health, or Epworth Camberwell. In these instances, pre-study screening will be done in person with the investigator. Participants will be supplied with the Patient Information and Consent Form (PICF) detailing the nature and purpose of the study. There will be an opportunity for participants to discuss the study with investigators as well as their treating medical clinician, and provide signed consent at the time. Alternatively, potential participants will be invited to consider their involvement in the study. Investigators can follow up with the latter post-discharge and if willing, these participants can return a signed Consent Form in a reply-paid envelope that they were previously provided.

Outpatients: In some cases, eligible participants will be identified post-discharge. In these instances, the investigator will call the participant to discuss the study and complete the pre-study assessment questionnaire. Participants who appear eligible and are interested in receiving more information will be sent a copy of the PICF and a letter summarising the study. The investigator will contact the participant again in 7-10 days to discuss the study further. In the initial phone call and in the letter, participants will be asked *not* to sign and return the consent form. If participants are interested in taking part in the study after this second phone call, they may sign the consent form at this time and send it back to researchers in a reply-paid envelope, or they will be asked to visit the Melbourne Brain Centre, Sunshine Hospital, or The Alfred Centre, to sign the consent form and begin the study.

The following are methodologies undertaken throughout the duration of the study.

III. Physical measurements

Body mass index and waist and hip circumference measures will be used to assess the body composition of the participants.

IV. Blood Collection for genotyping and biomarker analyses.

It is intended that blood samples will be collected within the same time period at every participant visit in an attempt to account for any biomarker circadian variation. Trained study investigators will collect a blood sample from participants. A total of 37 mls of whole venous blood will be collected at the baseline visit and 27 ml will be collected during the two follow-up visits.

Apolipoprotein E (APOE) genotyping: We will be determining the APOE genotype in our study population for three reasons. First, it allows us to investigate whether APOE status is a shared risk factor underlying stroke and dementia. Second, we need to know whether APOE is potential confound against the utility of exercise intervention on neurodegenerative processes. Third, given its strong relationship to Alzheimer's Disease, an inadvertent over-representation of APOE ε4 homozygosity would act as a potential confound for our study.

Two 5 ml vacutainer tubes of venous blood will be taken during the first, second, or third testing session (i.e., 2, 4, or 12 months post-stroke) for genetic analysis. One sample will be used for APOE genotyping and the second will be used for genetic analyses by the study investigators.

Participants will have the option of being informed about their APOE ε4 status. It will be explained to stroke participants that this may have been a risk factor for their stroke. Participants who have APOE ε4 homozygosity will be offered counselling via the Neurogenetics Clinic at Austin Health if they have further questions or concerns, or if their family members have questions.

APOE Analysis techniques: Genomic DNA (50 ng) is extracted using the Genomic-tip 20G (Qiagen, Hilden, Germany) and the APOE region of interest will be amplified with specific primers (Invitrogen, Carlsbad, CA). The resulting DNA fragment will be sequenced with BDV3.1 (Applied Biosystems, Foster City, CA) using the APOE-forward primer on an ABI 3130-xl genetic analyzer.

BDNF and HbA1c: Of the whole blood drawn, 9 mls will be collected in a plain tube, allowed to clot for at least 20 minutes at room temperature, and then centrifuged. The serum will be divided into 4 x 1.5 ml tubes for storage at -20°C until assayed. The remaining 18 mls will be collected in lithium heparin/EDTA tubes, stored on ice, and centrifuged within 15 minutes to separate the plasma. Plasma will be aliquoted into 8 x 1.5 ml tubes and storage at -80°C until assayed.

The samples will be split in two so that two separate freezers contain the samples. This is performed so that if for any reason one freezer has a fault that thaws the sample, a backup sample is still available for analyses. The freezers will both be locked. All sample tubes will only contain the ID number of the participant and date of collection and will contain no names.

Planned exploratory analysis will include BDNF and HbA1c assays on sample batches. A HbA1c tube is taken at each timepoint assessment alongside additional research blood samples. We anticipate that most samples will be collected at the baseline session by a

trained investigator or stroke research nurse at The Florey or Sunshine Hospital.

Inflammatory marker assays: Blood plasma samples will be transferred from The Florey (Melbourne Brain Centre) to Monash University for analysis by Dr. Barbara Cordoso and her laboratory team. This will include a multiplex panel of 6 biomarkers: IL-6, IL-1β, TNF-α, IL-8, IL-10, and IL-1ra (Method: Multiplex ELISA (Millipore). Monash University will be provided with the results of these analyses once they are completed.

It is possible that unused portions of blood samples collected in the current study will be tested for other biological markers at some stage in the future. However, such analysis is not an immediate focus of the study protocol. Any further analysis of blood samples not detailed in this Protocol will be raised with Austin Health HREC if/when that time arises.

V. Ambulatory Blood Pressure and ECG monitoring

24-hour ambulatory BP monitoring and 15-minute ECG-based heart rate variability for autonomic nervous system function assessment will be conducted for each assessment visit. Each participant will have a CardXplore monitor fitted for 24 hours. The CardXplore is a combined 24-hour ambulatory blood pressure and Holter ECG recorder. Trained staff will fit the monitor and explain the procedure to the participant. Analyses will be undertaken with the guidance of Associate Professor Christopher O'Callaghan in Clinical Pharmacology.

Circadian BP pattern classification will be based on the per cent decline in night-time BP compared with daytime BP. The normal ranges of ambulatory BP are defined as follows: daytime BP <135/85 mm Hg, night-time BP <120/75 mm Hg, 24-hour BP <130/80 mm Hg. For secondary hypotheses, statistical analysis will be performed using BP as a binary variable; i.e., abnormal BP, yes/no.

CardXplore monitors will be fitted during the first part of each timepoint so that the participant can return the monitor during the second part of each timepoint, which will minimise inconvenience to participants.

VI. MR Image Acquisition, Processing, and Analysis pipeline

High resolution structural magnetic resonance imaging (MRI) of study subjects will be acquired using a 3T clinical MR scanner. High resolution 1 mm isotropic T1-weighted 3D MP-RAGE, T2 FLAIR and T2 3D images will be acquired. Other images to be acquired in the same scanning session: Diffusion weighted image (DWI) to check for recent stroke; susceptibility weighted image (SWI) to check for microbleeds; a "free run" EPI scan (approximately 7 minutes) for connectivity analysis, and an arterial spin labelling sequence (approximately 10 minutes) to measure cerebral blood flow. Total scan time will be approximately 65 minutes.

<u>Recurrent stroke will be assessed on imaging characteristics at each MRI</u>: Intercurrent stroke between scanning intervals will be diagnosed by 2 neurologists on inspection of FLAIR, T1 and T2 images for stroke < 10 days old. DWI images will be inspected for recent stroke. These will be manually traced and masked for future analyses, and all stroke volume calculated for later use in exploratory analyses. For secondary hypotheses, presence of

recurrent stroke (clinical or silent) will be dichotomised as a binary variable; i.e., recurrent stroke: yes/no (see statistical analyses).

Structural analyses: cortical thickness and white matter hyperintensity volume: Cortical thickness will be mapped using the FreeSurfer software package for analysis of structural MRI [33]. Hippocampal volume estimation will be performed using Adaboost, which has been shown to have ICCs of 0.94-0.96 with manual tracing [34]. We will use QUANTA for WMH segmentation and estimation.

VII. Cognitive, Mood, Well-being, and Physical Activity assessment questionnaire/ tests

Participants will complete a neuropsychological assessment at each post-stroke time-point (Baseline, 4 months, 1 year). Seven cognitive domains (global cognitive ability, attention, processing speed, visuospatial ability, memory, language, and executive function) will be assessed using a neuropsychological battery developed from CANVAS that takes approximately 90 minutes [3]. The battery will include three computerised tests from the CogState Battery as well as the following standard paper-and-pencil tests:

Cognitive Domain	Cognitive task
Global Cognitive Ability	- Montreal Cognitive Assessment (MoCA) (original version at baseline; alternate versions at 4 and 12 months post-stroke)<br>- Alzheimer's Disease Assessment Scale-Cognitive Subscale (ADAS-Cog) (only administered at Baseline and 12 months post-stroke as no alternate versions are available, to counteract possible practice effects)
Attention	- Digit Span Task from the Weschler Adult Intelligence Scale-Fourth Edition (WAIS-IV)<br>- Trail-Making Test (TMT)<br>- Identification Task (Computerised CogState battery)<br>- One Back Task (Computerised CogState battery)
Processing Speed	- Digit-Symbol Coding Task from the WAIS-IV<br>- Detection Task (Computerised CogState battery)
Visuospatial Ability	- Rey Complex Figure (RCF) - Copy<br>- Cancellation Task from the WAIS-IV
Memory	- Hopkin's Verbal Learning Test – Revised (HVLT-R)<br>- Rey Complex Figure (RCF) - Recall
Language	- Verbal Fluency Task (VFT)<br>- Boston Naming Test (BNT)
Executive Function	- Clock Drawing Test (CDT)

All of these tasks have been designed for serial testing and are not subject to practice effects. In the case of the HVLT-R and Cancellation Task, alternate forms are available to use on repeat administrations. Performance on individual cognitive tests will be standardised using established norms, and domain scores will be calculated by averaging the standardised scores from each contributing test (e.g., digit span and digit symbol for the attention domain). In addition, the National Adult Reading Task (NART) will be used to estimate full-scale IQ; the Modified Rankin Scale (mRS) will be used to assess ability to function daily; the National Institute of Health Stroke Scale (NIHSS) will be used to assess neurological function; and the Fugl-Meyer Upper Limb Assessment will be used to provide a more detailed assessment of motor function, specifically for the upper limb and hand. The NART will be administered at one time-point, the mRS, Fugl-Meyer Assessment, and NIHSS will be administered at every time-point.

Assessment of mood will be completed using the Patient Health Questionnaire (PHQ-9) and the Generalised Anxiety Disorder 7-item scale (GAD-7). These questionnaires are based on criteria for the diagnosis of generalised anxiety and depression, as outlined in the Diagnostic and Statistical Manual-Fourth Edition (DSM-IV, no updated tests are yet available according to updated DSM-V criteria).

‘Quality of life’ will be quantified using the Assessment of Quality of Life (AQOL). Self-perceptions of fatigue will be recorded using the Fatigue Assessment Scale (FAS). Both of these scales have been developed for research projects and have been extensively used by the Investigators.

Subjective memory loss/improvement will be assessed at each time-point with the Memory Assessment Clinic-Questionnaire (MAC-Q), a validated and widely used 6-item questionnaire to measure self-report memory complaints over time.

Self-report physical activity will be assessed via the Physical Activity Scale for the Elderly (PASE), a validated and widely used questionnaire to track typical physical activity levels in an older adult population over a one-week period. We will administer the PASE in person at the 2-month, 4-month, and 12-month time-points, and over the telephone at 6, 8, and 10 months post-stroke. This will be administered to gauge activity levels outside of the intervention throughout the course of the study.

At the completion of the study, participants who request information regarding their cognitive test progress will be provided with a letter indicating whether they are performing at, below, or above cognitive norms standardised for age. This letter will also be accompanied by the flyer “*What Can I Do to Improve My Body and Brain Health?*”, which provides participants with information for how to improve their body and brain health, and encourages them to seek further referral advice from their local doctor should they have any concerns about their health, particularly regarding cognition.

VIII. Fitness test of aerobic capacity – VO_2 peak graded exercise test (GXT)

Aerobic capacity will be repeatedly assessed at each study visit via a submaximal, incremental exercise test. At the initial Baseline visit, information from the GXT will also be used to create each participant's graduated exercise programme.

All participants will undergo a VO_2peak graded exercise stress test to determine exercise capacity using a metabolic cart (the Oxycon mobile™, CareFusion). A submaximal GXT will be conducted at each of the three study visits to monitor the change in aerobic capacity. The GXT will take place at the Florey, Melbourne Brain Centre, Austin campus, or in the AIMSS facility at Western Health, under the supervision of a trained exercise professional. This person will not be involved with the exercise training as they are not blinded to participant status.

The following parameters will be recorded during the test: VO_{2peak} (volume oxygen uptake in ml/kg/min), Heart Rate (HR), Heart Rate Reserve (HRR, subtracting resting heart rate from the maximum heart rate measured during an exercise test), Oxygen Saturation, and Respiratory Rate (RR, number of breaths per minute). These parameters will enable us to derive VO_{2peak} as an estimate of aerobic exercise capacity. Blood pressure and ECG recordings will be monitored as markers of participant well-being.

A total body recumbent stepper will be used to perform the GXT, as outlined by Billinger 32]. This submaximal GXT protocol has been shown to be a valid measurement of VO_{2peak} [35], and prescribing cardiorespiratory exercise based on VO_{2peak} values is safe in a subacute stroke population with mild to severe post-stroke deficits [36].

Participants will be asked to maintain a step rate of 80 steps per minute on a recumbent stepper. The test will start at a power output of 15 watts and will be increased every 2 minutes by an amount that is predefined in the protocol (see Box 1, based on Billinger 2008). The amount will increase until completion or until volitional fatigue, or 85% of age predicted heart rate was achieved (0.85[220-age]). HR will be recorded every 10 seconds at the end of the second and third minute. If the change in HR between the second and the third minute is less than 5 bpm then the resistance will be moved to the next stage. If it is over 5 bpm an additional minute will be performed at the same stage to make sure steady state will be reached. VO_2peak will be determined by imputing HR and workload into the following equation VO_2peak (ml.kg.min) = 125.707 + (-0.476)(age) + (7.686)(sex [0= female; 1= male]) + (-0.451)(weight in Kg) + (0.179)(Watts end_submax) + (-0.415)(HRend_submax).

**Stage**	**Resistance (W)**	**Stepping cadence (steps x min)**	**Duration (min)**
Load 1	25	80	2
Load 2	40	80	2
Load 3	55	80	2
Load 4	70	80	2
Load 5	85	80	2
Load 6	100	80	2
Load 7	115	80	2

Load 8	130	80	2

Based on exercise protocol described in Billinger et al. [35].

The GXT will be stopped using the safety criteria of exercise testing according to the American College of Sports Medicine (ACSM) exercise testing and prescription guidelines [37].

<u>Absolute stopping criteria</u>:
The exercise stress test will be stopped and the participant will be excluded if any of the following occur:

- drop in systolic BP of >10 mm Hg from baseline BP despite an increase in workload when accompanied by other signs of ischemia.
- Moderately/ severe angina (defined as 3 on the ACSM standardised rating scale),
- increasing nervous system symptoms (e.g. ataxia, dizziness, or near syncope),
- signs of poor perfusion (cyanosis or pallor),
- subject desire to stop,
- sustained ventricular tachycardia, ST elevation (+1.0 mm) in leads without diagnostic Q waves (other than V1 or aVR).

We will also use the following relative criteria. If the participant meets one of the relative criteria below, we will consult the treating clinician to determine if the participant is medically sound to be included in the trial and perform the exercise session at the planned intensity.

- drop in systolic BP of >10 mm Hg from baseline BP despite an increase in workload in absence of other signs of ischemia
- ST or QRS changes such as excessive ST depression (>2 mm horizontal or down-sloping ST-segment depression) or marked axis shift
- Arrhythmias other than sustained ventricular tachycardia, including multi focal PVCs, triplets of PVCs, supraventricular tachycardia, heart block, or brady-arrhythmias
- Fatigue, shortness of breath, wheezing, leg cramps or claudication
- Development of bundle-branch lock or intra-ventricular conduction delay that cannot be distinguished from ventricular tachycardia
- Increasing chest pain

Alongside the physiological recordings, the Borg Rating of Perceived Exertion (RPE) [38] will be used in the Fitness test as a subjective reporting measure of exercise difficulty, in order to help moderate activity intensity.

At the completion of the study, participants who request information regarding their Fitness test progress will be provided with a letter indicating whether they are performing at, below, or above the average for their age range, compared to people who have not experienced a stroke. This letter will also direct participants to speak to their local doctor about possible referral to an Exercise Physiologist or Physiotherapist, should they express interest in improving their physical fitness subsequent to participation in the study.

IX. Physical activity monitoring

Physical activity and energy expenditure will be monitored by an Actiwatch Spectrum Plus (Koninklijke Philips, Amsterdam, Netherlands). The Actiwatch is a small, unobtrusive electronic physical activity monitor on an adjustable wrist strap that is worn on the left wrist. If participants have left-arm paresis the monitor will be fitted to the right wrist. Participants will be asked to wear the waterproof Actiwatch 24 hours per day for 7 days. In previous studies, conducted by the current Investigators, involving the use of Actiwatches for the duration specified there have been no complaints or concerns raised by participants.

The Actiwatch will be fitted onto participants at each of the three timepoints throughout the study, after the MRI scan and cognitive testing has been completed. Following the 7 day monitoring period, participants will be asked to return the de-identified Actiwatch in a de-identified pre-paid envelope to the study investigators.

In addition, participants will be asked to keep a daily activity diary that records participant activity levels between assessment visits. Such diaries are common place as a part of general post-stroke rehabilitation programmes and this is not expected to be an inconvenience for participants.

Time spent active will be used as a co-variate for data analysis.

X. Sleep monitoring

The Actiwatch Spectrum Plus (Koninklijke Philips, Amsterdam, Netherlands) has the capacity to record objective sleep activity, therefore two measures will be used to track subjective assessment of sleep during the same three time-points as the Actiwatch use (as above).

Use of a Sleep Diary is common practice in sleep research during the period of wearable sleep trackers (such as the Actiwatch) in order to corroborate objective measures of sleep. Therefore, an adapted Sleep Diary from the widely used Core Consensus Sleep Diary [39] will be used during the one week of Actiwatches to capture subjective bedtime, sleep time, nighttime awakenings, and morning rise time. 2. The Pittsburgh Sleep Quality Index (PSQI) [40] questionnaire is a retrospective self-report 10-item questionnaire that invites participants to rate their sleep quality and sleep disturbances over a one-month period. The PSQI yields a global score of sleep quality across seven domains: subjective sleep quality, sleep onset latency, sleep duration, sleep efficiency, sleep disturbances, use of sleeping medications, and daytime dysfunction, with a global score of >5 indicating poor sleep. The PSQI is considered the standard subjective sleep questionnaire and widely used and validated within the research community, found to have high internal consistency and used with both older adults and stroke patients [27, 41, 42].

The Sleep Diary and sleep questionnaire will be returned with the Actiwatch at each assessment. Completion time for both the subjective measures of sleep is expected to take between 10-30 minutes (in total), per each assessment time-point.

Changes in sleep (e.g., significantly increased or decreased total sleep time, increased sleep onset latency, decreased sleep efficiency, increased wake after sleep onset) will be used in analyses as a moderator for improvement in brain health.

XI. Stool sample collection for microbiome analysis

At each timepoint, participants will be provided with a specimen kit to take home and asked to collect a stool sample. They will be asked to store the sample in the freezer until it can be returned to the facility. Participants will also receive a thermo bag to place the sample in for preservation purposes when returning it to the facility. This will be completed after each timepoint. In the case of Visit 2 (2-month timepoint), participants will complete the stool sample before they begin the exercise intervention.

The samples will be used to determine the effects of exercise on gut microbiome composition, and relationships with cognitive and brain volume change after stroke.

A portion of samples from each participant will be transferred from The Florey or Sunshine Hospital (depending on site) to Monash University for analysis by A/Prof Francine Marques and her laboratory team who will prepare stool samples using reagents and arrange for microbial sequencing.

It is possible that unused portions of stool samples collected in the current study will be tested for other biological markers at some stage in the future. Any further analysis of stool samples not detailed in this Protocol will be raised with Austin Health HREC if/when that time arises.

XII. Dietary intake monitoring

Dietary intake will be recorded by participants using the Research Food Diary app (Xyris Software (Australia) Pty Ltd.) At the 2 month timepoint, participants who have a smart phone (Android or iOS) will receive information on the app's basic functions and how to record dietary intake on the app. Participants will complete a 3-day food diary (2-week days and 1 weekend) in the week following each timepoint: 2 months, 4 months, and 12 months. At each timepoint, participants will use the feature "share" in the app to send their 3-day food diary to the study nutritionist, who will check the food diary for completion. If any inconsistency is observed, the nutritionist will advise the Project Coordinator, who will contact the participant by phone, and corrections will be performed if necessary. Nutrient analysis will be undertaken using an Australian food composition database, the FoodWorks Professional (v8) software.

Qualitative sub-study – The experience of post-stroke exercise therapy

All 120 participants in the study will be invited to participate in a qualitative descriptive sub-study that explores the experience of partaking in post-stroke home-based exercise. A semi-structured interview will be used to capture the information. Understanding the barriers and facilitators of participant engagement in post-stroke home-based exercise will assist in translating the knowledge harnessed from this project into clinical practice when prescribing post-stroke treatment and care. It will also assist in the development and formulation of future such research.

Exclusion criteria: Stroke participants with severe speech impairments or with English as their second language and which prevents effective communication (eg. severe aphasia) as determined by their treating speech pathologist will be excluded. An inability to effectively

communicate with or without an interpreter will exclude the participant from this sub-study but will not prevent participation in the overall trial.

Data collection: Qualitative data will be collected from eligible participants at the 1 year review session. Participants without stroke-related communication impairments will be asked to partake in a semi-structured in-depth face-to-face interview from a trained Associate Investigator.

Semi-structured interview guide sheets will be used to ensure all areas of interest are covered, but will allow for probing or additional questions if interesting concepts emerge. An example of the interview guide has been provided.

All interviews will be digitally audio-recorded with the participants' consent. During and/or after the interviews, field notes will be taken to add context and richness to the data collected. For example this may include notes on the time and place of the interview, whether family members are present, etc.

6. Study Population

a. Recruitment Procedure

120 newly acquired ischaemic stroke participants (i.e., <2 months post-ischaemic stroke) will be recruited from the Austin Health, Eastern Health, Western Health, and Epworth Camberwell Stroke Units and followed longitudinally. All ischaemic stroke subtypes will be included. Several methods will be used to recruit participants over the 3 year recruitment period of the study:

a) The Principal Investigator (Prof Amy Brodtmann) holds clinical appointments at both Austin Health and Eastern Health Stroke Units.
b) Senior clinicians on all Stroke Units will be notified of the study and asked to identify potentially suitable participants.
c) Study investigators will attend ward rounds and clinical meetings to identify potentially suitable participants.

In each case of the above, potential participants will be approached with the guidance of Prof Amy Brodtmann or the senior inpatient ward clinician. The study investigators will speak to potential participants and provide them with verbal and written information on the study.

Participants will be screened to ensure that they are cognitively able to participate in the study and have no contraindication to the MRI. The investigators will screen potential participants using a short, semi-structured questionnaire. If the recruiter is unsure whether the potential participant has the cognitive capacity to participate, the opinions of the study neuropsychologist will be obtained.

Demographic and medical information including age, gender, medication status, side and site of stroke and admission stroke severity (National Institutes of Health Stroke Scale, NIHSS) and functional status (modified Rankin Score, mRS) will be obtained.

b. Inclusion Criteria

120 participants who recently acquired ischaemic strokes (i.e., ischaemic stroke <2 months) will be recruited. All ischaemic stroke subtypes will be included.

Participants will be included if the following eligibility criteria are met during the initial screening:

- Ischaemic clinical stroke patient (first or recurrent stroke) aged greater than 18 years;
- Cardiac function not precluding exercise intervention;
- Premorbid mRS<3, denoting no, mild or moderate disability; and
- Able to give consent; and
- Able to nominate an emergency contact person.

c. Exclusion Criteria

Participants will be excluded if the following eligibility criteria are not met during the initial screening:

- Significant medical co-morbidities (e.g., severe cardiac disease) and/or musculo-skeletal injuries precluding participation in exercise intervention, or making survival for 1 year post-stroke unlikely;
- Pregnancy;
- Normal exclusion criteria for MRI; e.g., implanted metal, severe claustrophobia;
- Pre-existing dementia
- mRS >3.*

* In order to have a cohesive cohort for this study, we have elected not to include participants with a premorbid diagnosis of dementia or cognitive decline.

d. Consent

Written informed consent will be obtained from participants via a Participation Information and Consent Form (PICF). The PICF will detail the purposes of the project, issues surrounding confidentiality and the storage and sharing of their data, potential benefits and risks associated with their participation, and other information required for participants to make an informed decision about their participation.

A copy of the PICF will be provided to each participant to read through (i.e., in person while they are inpatients or via mail post-discharge), and they will be given time to consider their participation in the study within the 2-month post-stroke timeframe. As we outlined above in Section 6 (Recruitment Procedure), participants will be screened to ensure that they have the cognitive capacity to provide informed consent – that is, we do not intend to ask their spouse or next-of-kin to provide informed consent on their behalf at recruitment.

For those participants who do not provide signed consent during their inpatient admission, a reply-paid envelope will be given for them to return the Consent Form once they have made a decision. If the Consent Form is not received from potential participants within the 2 month post-stroke time period they will be contacted via phone by one of the study investigators to ask if they have any further questions about the study.

As detailed in Section 5d (II), some participants will be invited to take part in the study after they have been discharged. The investigator will call the participant to discuss the study and complete the pre-study assessment questionnaire. Participants who appear eligible and are

interested in learning more about the study will be sent a copy of the PICF. They will be asked not to sign and return and consent form. The researcher will call the participant in 7-10 days to discuss the study further, and if interested, the participant may sign the consent form at this time and send it back to researchers in a reply-paid envelope, or the researcher will organise a visit to the MBC, Western Health, or The Alfred Centre to sign the consent form. Within the PICF document is a separate Consent Form for the collection of blood for the purpose of genetic analysis and biomarker analyses. Participants will have the option of Consenting to their blood being utilised for either or both of these purposes. Within the PICF is also a separate Consent Form for the collection of a stool sample for the purpose of microbiome analyses. Participants will have the option of Consenting to provide a sample for this purpose. Furthermore, participants are still able to partake in the wider study if they do not wish for blood or stool samples to be taken or be used for any of the aforementioned analyses.

In cases where a study partner is required to attend the exercise sessions, they will need to sign a separate consent form themselves.

7. Participant Safety and Withdrawal

a. Risk Management and Safety

There is potential for participants to experience claustrophobia and discomfort from noise in the MRI scanner; potential for discovering new/confronting information on MRI; cardiovascular events or respiratory events as a result of physical exercise; and physical injury as a result of physical exercise. Each of these risks are addressed in turn below:

Claustrophobia and discomfort from the noise of the MRI scanner

There is potential for participants to experience claustrophobia and discomfort from the loud banging noise in the MRI scanner. These risks are outlined in the PICF.

To minimize the likelihood of an adverse reaction during the scan, participants will be asked whether they feel uncomfortable in tight spaces (e.g., elevators), or if they tend to experience discomfort in noisy environments, *before* they consent. If they indicate that they are likely to experience an adverse reaction to the scan, or if the investigator believes that this is likely to happen, it will be recommended to participants that they do not take part in the study.

Participants will be able to communicate with the radiographers at all times during the scan. If they experience any discomfort, they can notify the radiographers by pressing a buzzer, and the scan will be discontinued immediately. Participants will be able to watch movies and listen to music.

Potential for discovering new/confronting information on MRI Brain

We are obligated to notify all participants of the results of their MRI Brain scan. The investigators will explain that there is small chance a significant abnormality, not previously known which requires further investigation, might be detected on the MRI scan. If an abnormality is detected on a scan, Prof Amy Brodtmann will place a call to the participant and through their GP refer them onto appropriate members of a clinical team who are not

part of this project. Prof Brodtmann will also offer them counselling or other appropriate support by staff members, who, again, are not part of the project.

Cardiovascular events or respiratory events as a result of physical exercise

Although rare, two common cardiac risks include myocardial ischemia and different forms of arrhythmias. It is estimated that for adults without existing heart disease, the risk of a cardiac event or complication ranges between 1 in 400,000–800,000 hours of exercise. For patients with existing heart disease, an event can occur an average of once in 62,000 hours [43]. The exercise tests and training also involve a risk of vasovagal episodes.

To mitigate the risk of a vasovagal episode or any other significant adverse cardiac event, all participants must be cleared by a consulting physician before commencing the exercise test and exercise training intervention. The exercise testing and training will be in accordance with current guidelines from the ACSM Exercise Testing and Prescription (ACSM, 2013). Dr Liam Johnson (Associate Investigator) is experienced in conducting exercise tests and prescribing exercise and will be responsible for training staff involved in the exercise component of the study.

This study is employing a submaximal, graded exercise test, and the exercise intervention includes CRF training at 50 to 75% of the participant’s maximal aerobic capacity. It is not anticipated that the participants will be exercising at an intensity that is likely to induce significant adverse events.

Irrespective, the participant will be closely monitored at all times during the exercise test and during the exercise intervention. Participants will be required to nominate somebody the research team can contact in case of emergency during the exercise intervention. This person may also be involved as a study partner and need to attend each of the exercise intervention sessions if the Exercise Professional deems it necessary. All investigators involved in the exercise testing are required to maintain current first aid qualifications.

Participants will be educated about the procedure to follow should any concerning cardiovascular signs or respiratory signs occur off-site (i.e., not at the Austin Health campus, Eastern Health campus, Western Health campus, or The Alfred Centre) whilst enrolled in the study. Participants will be provided with a ‘Participant Adverse Event Notification Form’ to document events and notify Investigators.

Physical injury as a result of physical exercise

All exercise procedures involve some risk to participants. It is possible that the participants will experience muscle soreness and stiffness as a result of completing the exercise tests and training. Such feelings are likely to subside within 24 to 48 hours after the exercise and leave no sustained muscle damage.

If participants raise concern about a physical injury sustained while participating in the study then these will be assessed by Dr Liam Johnson (Senior Research Exercise Physiologist) or another member of the research team with appropriate training (medical doctor [e.g., Prof Amy Brodtmann or Professor Vincent Thijs] or physiotherapist [e.g., Prof. Julie Bernhardt]).

Appropriate diagnosis and treatment will be arranged, without monetary cost to the participant, through the health service from which they were recruited.

Blood sampling

Blood donations are generally very safe and side effects are rare. Pain and bruising may occur at the needle placement sites. As with other kinds of blood drawing, temporary lowering of the blood pressure may develop, and light-headedness, dizziness and even fainting may result. Trained study Investigators or stroke research nurses at The Florey, Sunshine Hospital, or The Alfred Centre will perform the blood draw and preliminary processing. Sterilized, single-use equipment will be used to draw blood is and disposed of following a donation procedure. Blood drawing equipment is never in contact with blood from another individual. No blood products are given during these procedures. Medical doctors are available at all times during the donation procedure to provide short-term medical care for any complications or reactions resulting from donation procedure.

b. Adverse event management

Investigators will complete an 'Adverse Event Case Report Form' to detail and monitor any cases of adverse events occurring within the study.

At each exercise session between two and four months post-stroke, and at the second and third visits (four and 12 months post-stroke, respectively), participants will be assessed for possible Adverse Events (AEs). AEs will be reported as they occur in the participant's CRF.

AEs that meet the criteria for serious, are considered Serious Adverse Events (SAEs) and will be reported for each participant for the duration of their participation in the study (i.e., between two months and 12 months post-stroke). The investigator or designee will ask the participant non-leading questions in an effort to detect adverse events, and serious adverse events.

Definition of an Adverse Event (AE)

An Adverse Event (AE) is any untoward medical occurrence in any participant involved in the study. It does not necessarily have to have a causal relationship to the study intervention.

Examples of an AE include:

- Exacerbation of a chronic or intermittent pre-existing condition including either an increase in frequency and/or intensity of the condition.
- New conditions detected or diagnosed after involvement in the study even though it may have been present prior to the start of the study.

Definition of a Serious Adverse Event (SAE)

- A serious adverse event is any AE that:
- a) results in death
- b) is life threatening
    - Note: The term 'life-threatening' in the definition of 'serious' refers to an event in which the participant was at risk of death at the time of the event. It does not refer to an event, which hypothetically might have caused death if it were more severe.

- c) requires hospitalisation or prolongation of an existing hospitalisation.
    - Note: In general, hospitalisation signifies that the participant has been detained (usually involving at least an overnight stay) at the hospital or emergency ward for observation and/or treatment that would not have been appropriate in the physician's office or out-patient setting. Complications that occur during hospitalisation are AEs. If a complication prolongs hospitalisation or fulfils any other serious criteria, the event is serious. When in doubt as to whether 'hospitalisation' occurred or was necessary, the AE will be considered serious.
    - Hospitalisation for elective treatment of a pre-existing condition that did not worsen from baseline is not considered an AE.
- d) results in disability/incapacity
    - Note: The term disability means a substantial disruption of a person's ability to conduct normal life functions. This definition is not intended to include experiences of relatively minor medical significance such as uncomplicated headache, nausea, vomiting, diarrhoea, and influenza, which may interfere or prevent everyday life functions, but do not constitute a substantial disruption.

Medical and scientific judgement will be exercised in deciding whether reporting is appropriate in other situations, such as important medical events that may not be immediately life-threatening or result in death or hospitalisation, but may jeopardise the participant or may require medical or surgical intervention to prevent one of the other outcomes listed in the above definition. These will also be considered serious. Examples of such events are invasive or malignant cancers, intensive treatment in an emergency room or at home for allergic bronchospasm, blood dyscrasias or convulsions that do not result in hospitalisation, or development of drug dependency or abuse.

The investigator will exercise his or her medical and scientific judgement in deciding whether an abnormal laboratory finding or other abnormal assessment is clinically significant.

Time Period, Frequency, and Method of Detecting AEs and SAEs

All AEs and SAEs will be recorded from the time of consent until the final follow up visit at one year post-stroke. Each participant will be monitored regularly by the investigator and study personnel for events occurring throughout the study.

During the study, the investigator will enquire about AEs by asking the following non-leading questions:

"How are you feeling?"

At subsequent scheduled intervals participants will be asked:

"Since you were last asked, have you felt unwell or different from usual?"

Recording of AEs and SAEs

When an AE/SAE occurs, the investigator will review all documentation (e.g. hospital progress notes, laboratory, and diagnostic reports) relative to the event. The investigator will then record all relevant information regarding an AE/SAE in to the CRF.

For each reportable event, start and stop dates, action taken, outcome, intensity and relationship to study treatment (causality) will be documented. If an AE changes in frequency or intensity during a study, a new entry of the event will be made.

The investigator will attempt to establish a diagnosis of the event based on signs, symptoms, and/or other clinical information. In the absence of a diagnosis, the individual signs/symptoms will be documented.

All details of any treatments initiated due to the adverse event will be recorded in the participant’s notes and the CRF.

The investigator will notify the Ethics Committee of any SAEs once per year in the annual progress report.

Assessment of Intensity

The investigator will make an assessment of intensity for each AE and SAE reported during the study. The assessment will be based on the investigator’s clinical judgement and assigned to one of the following categories:

Mild: An event that is easily tolerated by the participant, causing minimal discomfort and not interfering with everyday activities.

Moderate: An event that is sufficiently discomforting to interfere with normal everyday activities.

Severe: An event which is incapacitating and prevents normal everyday activities.

An AE that is assessed as severe should not be confused with an SAE. Severity is a category utilised for rating the intensity of an event; and both AEs and SAEs can be assessed as severe. An event is defined as “serious” when it meets one of the pre-defined outcomes as described.

Assessment of Causality

A blinded assessor will assess the relationship between study treatment and the occurrence of each AE/SAE. The blinded assessor will use clinical judgment to determine the relationship. Alternative causes, such as natural history of the underlying diseases, concomitant therapy, other risk factors, and the temporal relationship of the event to the treatment period will be considered.

The causal relationship to the study treatment will be assessed using the following classifications:

Not Related	In the Assessor’s opinion, there is not a causal relationship between the study product and the adverse event.

Unlikely — The temporal association between the adverse event and study treatment is such that the study treatment is not likely to have any reasonable association with the adverse event.

Possible — The adverse event could have been caused by the study treatment.

Probable — The adverse event follows a reasonable temporal sequence from the time of study treatment, abates upon discontinuation of treatment and cannot be reasonably explained by the known characteristics of the study participant's clinical state.

Definitely — The adverse event follows a reasonable temporal sequence from the time of study treatment start or reappears when study treatment is reintroduced.

Assessment of Expectedness

Expected — An adverse event, the nature or severity of which is consistent with the clinical condition of the participant.

Unexpected An adverse event, the nature or severity of which is not consistent with the clinical condition of the participant.

Follow-up of AEs and SAEs

All AEs and SAEs documented at a previous visit/contact and are designated as ongoing, will be reviewed at subsequent visits/contacts.

All AEs and SAEs will be followed until resolution, until the condition stabilises, until the event is otherwise explained, or until the participant is lost to follow-up.

New or updated information will be recorded on the originally completed SAE form, with all changes signed and dated by the investigator.

c. Handling of Withdrawals

If a participant experiences some distress as a result of participation in the study, and decides to withdraw, we will offer them counselling or other appropriate support by staff who are not members of the research team. No follow-up is required if a participant decides to withdraw because of a lack of motivation or interest.

The investigator will inform each participant *prior to consenting* that data collected about them up to the point of their withdrawal will be kept for the length of time specified by department protocol (i.e., 7 years for demographic and cognitive data; 10 years for blood samples), and will be included in all analyses. This is specified in the PICF and will be highlighted by the recruiters. However, withdrawals will be handled on a case-by-case basis – if a participant would prefer that we destroy their data and/or exclude their data from any analysis, we will certainly consider their request. If a participant is unable to be contacted, their data will be kept and included in all analyses.

We will document in the research record each instance of a participant's withdrawal from the study, along with the following details: whether the withdrawal was initiated by the participant; the reason for their withdrawal, if known; and whether a request has been made to withdraw their data.

d. Replacements

As discussed below in Section 8.a (Power Calculations), we expect around 30% attrition due to death or non-participation (e.g., unable to participate in testing due to new pacemaker/implanted metal/other medical issue, lost to contact, not interested). This has been taken into account in our sample size estimation and power analysis, and so, the statistical significance of the sample size is unlikely to be affected by participant withdrawal.

8. Statistical Methods

a. Sample Size Estimation & Justification

We aim to recruit 120 participants who have experienced ischaemic stroke in the past 2 months. We calculate 60 participants per group are required. We calculated our sample size (see Section 8.b.) in order to include sufficient participant numbers for estimation of the primary endpoint: brain volume at 4 months post-stroke.

Methods of participant recruitment are detailed in Section 6.a.

b. Power Calculations

The total sample size of 120 participants will yield 80% power to detect a difference corresponding to a medium to large effect size on brain volume measures, assuming the standard settings of 2-tailed significance, alpha=0.05, and d=0.6. This effect size is reasonable, given that in our CANVAS pilot data, we reported hippocampal and total brain volume loss of -3% and -1.5% respectively over one year, in contrast to the control change of -0.4% and -0.6% over the same time period.

We have increased the sample size by 30% to cover attrition over the 12 month period and the possibility of non-evaluable scans (e.g., movement, etc.) giving n=60 per group.

c. Statistical Methods To Be Undertaken

The analysis can be considered in 3 parts, aligned with primary and secondary hypotheses:

i. ***Primary outcome and hypothesis: Comparing brain volumes between stroke participant groups:*** Primary outcome will be analysed on an intention to treat basis, The difference in brain volume from baseline to 4 months will be analysed using an ANCOVA model with exercise intervention group as a factor (e.g., Physical fitness training vs. Balance training groups), with baseline brain volume and functional status as co-variants (dichotomised as mRS = 0-1, 2 and 3).

ii. ***Secondary outcome and hypotheses: Correlating brain volume with blood pressure control and incident stroke:*** Secondary outcomes will be analysed using appropriate repeated measures random effect logistic regression models with dichotomised hypertension status or silent/recurrent brain infarction (as defined in methods) as dependent variables, treatment group as independent variable, and functional status dichotomized as a covariate (as above). The initial regressive analysis will examine the

differences between participants with good and poor blood pressure control, using this as a binary measure. The presence of incident subclinical stroke will also be included as a binary variable. In the regional volumetric analyses, total brain volume, regional white matter, cortical and subcortical grey matter volume changes will be modelled using this design.

iii. ***Exploratory analyses: Independent effects of exercise on blood pressure, incident stroke, diastolic cardiac indices, and aerobic capacity:*** Exercise effects on end-organ function and fitness (i.e., aerobic capacity) are likely dissociable, however emphasise that this component is exploratory. For the purposes of these analyses, we define each of our end-organ metrics as: brain volume, cognitive function, incident stroke, diastolic cardiac indices, aerobic capacity (VO_2max), sleep duration, onset latency, bedtime/rise time variability (minutes), and sleep efficiency (%). Exploratory analysis will be conducted using appropriate regression models to investigate the associations between BP, incident stroke, diastolic cardiac indices, aerobic capacity. In addition, mediation analyses will be conducted to explore the possible mediating role of sleep in preservation of brain volume.

d. Qualitative Analysis

Data management

NVivo 10 software (or similar) will be used to assist data management. Digitally recorded interviews will be professionally transcribed verbatim and checked against the recordings and corrected for accuracy. Prior to analysis, all transcribed information that may identify an individual or a hospital (for example names will be replaced with assigned codes).

Data analysis

Qualitative descriptive content analysis will use a six step strategy [44] including: identifying and coding all meaningful text units from interview data and field notes; recording insights and reflections on the data; sorting the data to identify similar features, phrases, concepts; constant comparison to look for commonalities and differences among the data; deciding on generalisations or themes that hold true for the data and/or for particular participant sub-groups.

To enhance trustworthiness of the findings, this analytical process will be undertaken by two researchers who will familiarise themselves with the data through repeated reading of the transcripts and will undertake the coding step independently. For the latter iterative steps of analysis the researchers will work collaboratively using discussion to reach consensus.

Thematic saturation for a particular subgroup will provide confidence that our data is representative, and will be considered to have occurred if no new themes emerge in the final three interviews analysed.

9. Storage of Blood,Tissue and Stool Samples

a. Details of where samples will be stored, and the type of consent for future use of samples

Participants will be asked to provide 10ml of venous blood at their first, second, *or* third visit for APOE genotyping and analysis of other genetic and blood markers. They will provide an additional 27ml of venous blood at each study visit. Each sample will be assigned a unique identifier and will be locked in a storage freezer.

Participants will be asked to provide a stool sample at the 2-, 4-, and 12-month timepoints for the purpose of analysing the gut microbiome. Each sample will be assigned a unique identifier and will be locked in a storage freezer.

Consent will be obtained separately for blood and stool samples – that is, participants can refuse to provide either or both of the samples, and still take part in the study. Samples will be stored for future genetic or biomarker analyses for 10 years. After this time, they will be destroyed as per department protocol for human samples. If participants decline to consent for use in future research, the sample will be disposed of after a year following completion of the research.

10. DATA SECURITY & HANDLING

a. DATA COLLECTION

A Case Report Form (CRF) will be completed for each study participant summarising all clinical screening and study data. Participants will only be referred to in the CRF by their numerical identifier in order to retain confidentiality. All data entered on the CRF will have supporting source data located at the study site in the participant’s medical record.

b. DETAILS OF WHERE RECORDS WILL BE KEPT & HOW LONG WILL THEY BE STORED

c. *ANY IDENTIFIABLE DOCUMENTS WILL BE KEPT FOR 7 YEARS IN PROF BRODTMANN’S LOCKED OFFICE IN A LOCKED BUILDING (I.E., ALFRED CENTRE, MONASH UNIVERSITY) OR IN AN UNMARKED LOCKED FILING CABINET ON THE FOURTH FLOOR OF THE AIMSS FACILITY(FOR WESTERN HEALTH PARTICIPANTS). AFTER THIS TIME, PAPER COPIES WILL BE SHREDDED AND DISPOSED IN CONFIDENTIAL DOCUMENT PAPER RECYCLING. COMPUTER DISCS WILL BE ERASED, ALONG WITH BACK UP FOLDERS (HARD-DRIVES).* CONFIDENTIALITY AND SECURITY

The investigators intend to protect participants’ anonymity and the confidentiality to the fullest possible extent, within the limits of the law. Each participant will be assigned a numerical identifier and their name and contact details will be kept in a separate file from any data that they supply. In any publication and/or presentation arising from this research project, information will be provided in such a way that the participant cannot be identified. Data will be presented and discussed in terms of participant groups, not individual participants.

d. ANCILLARY DATA

MRI scans, if used in publications, will have all text details removed and consist of brain images only. It should be noted that all images acquired on the 3T BRI Siemens scanner contain no identifiable information, as they are produced in DICOM format which only allows the image file. Staff will assign each participant a unique numerical identifier, and only this is tagged to the participant's file, but is not present on any image once converted into a viewable mode. Scans will be kept on password-protected server, separate to any cognitive data that participants provide. No films will be printed. Scans will be stored on the server for 7 years, after which time they will be deleted.

Voice recordings as a part of the qualitative interview will also be kept on a password-protected server, denoted only by participant number, and separate to any other participant information.

11. REFERENCES

1. Brodtmann, A., et al., J Neurol Sci, 2012. 322(1-2): p. 122-8.
2. Brodtmann, A., et al. Brain atrophy rates are not affected by thrombolysis 3 months after MCA ischemic stroke. in International Stroke Conference. 2015. Nashville: Stroke.
3. Brodtmann, A., et al., Int J Stroke, 2014. 9(6): p. 824-8.
4. Iadecola, C., Hypertension, 2014. 64(1): p. 3-5.
5. Taylor, C., et al., J Am Geriatr Soc, 2013. 61(9): p. 1489-98.
6. Wolfson, L., et al., J Gerontol A Biol Sci Med Sci, 2013. 68(11): p. 1387-94.
7. White, W.B., et al., Am Heart J, 2013. 165(3): p. 258-265 e1.
8. Jack, C.R., Jr., et al., Lancet Neurol, 2010. 9(1): p. 119-28.
9. Jack, C.R., Jr., et al., Brain, 2009. 132(Pt 5): p. 1355-65.
10. Sona, A., et al., Int Psychogeriatr, 2012. 24(2): p. 197-204.
11. Tolppanen, A.M., et al., Alzheimers Dement, 2014.
12. Cumming, T., et al., Physical activity is associated with cognition at 3 months post-stroke, in Stroke Society of Australasia2014: Hamilton Island, Australia.
13. Bejot, Y., et al., PLoS One, 2011. 6(12): e29405.
14. Rodier, M., et al., PLoS One, 2015. 10(10): e0410668.
15. Sawrdfager, W., et al., Brain Behav Immun, 2011. 25(6): p. 1264-1271.
16. Marcus, R.L., et al., Phys Ther, 2008. 88: p. 1345-1354.
17. Myint, P.K., et al., Stroke, 2007. 38: p. 271-275.
18. Farrer, L.A., et al., Jama, 1997. 278(16): p. 754-764.
19. de Leeuw, F-E., et al., Stroke, 2004. 35: p. 1057-1062.
20. Knopman, D.S., et al., Alzheimers Dement, 2009. 5: p. 207-214.
21. Manning, E.N., et al., PLos One, 2014. 9(5): e97608.
22. Buxton, O.M. & Marcelli, E., 2010. Soc Sci Med., 71: p. 1027–36.
23. Koskenvuo, M., et al., British Medical Journal (ClinicalResearch Ed.), 1987. 294(6572), 643.
24. Bassetti, C.L., et al. Milanova, M., & Gugger, M.,Stroke, 2006. 37: p. 967–972.
25. Lim A.S.P, et al., Sleep 2013. 36: p.1027–1032
26. Sexton, C.E., et al., Neurology, 2014. 83(11): p. 967-973
27. Kim, J., et al., 2015. Ann Rehabil Med., 39(4): p. 545–552.
28. Zhang, S., et al., Behavioural Neurology, 2014(June 2011).

29. De Brujin, R. F., et al., BMC Med., 2015. 13(132). doi:10.1186/s12916-015-0377-5
30. Ashby-Mitchell, K., et al., Alzheimers Res. Ther. 2017. 9(11). doi:10.1186/s13195-017-0238-x
31. Jacka, F. N., et al., BMC Med., 2015. 13(215). doi:10.1186/s12916-015-0461-x
32. Gubert, C., et al., Neurobiol Dis., 2019. 16(134). doi:10.1016/j.nbd.2019.104621
33. Fischl, B., et al., Proc Natl Acad Sci U S A, 2000. 97(20): p. 11050-5.
34. Morra, J.H., et al., IEEE Trans Med Imaging, 2010. 29(1): p. 30-43.
35. Billinger, S.A., et al., Phys Ther, 2008. 88(10): p. 1188-1195.
36. Billinger, S.A., et al., J Neurol Phys Ther, 2012. 36(4): p. 159-165.
37. American College of Sports Medicine. ACSM's Guidelines for Exercise Testing and Prescription In: Pescatello L, editor. 9 ed2013.
38. Borg, G.A. Med. Sci. Sports Exerc, 1982. 14(5): p. 377-81.
39. Carney, C. E., et al. Sleep, 2012, *35*(2): p. 287–302.
40. Buysse, D. J., et al., Psychiatry Research, 1989. 28(2): p. 193 -213.
41. Cole, J.C., et al., Sleep, 2006. 29(1): p. 112-116.
42. Beaudreau, S.A., et al., Sleep Med, 2012. J3(1): P. 36-42.
43. Myers, J. Circulation, 2003. 107(1): e2-5.
44. Miles, M. Qualitative data analysis-An expanded sourcebook. 2nd Edition. Thousand Oaks: SAGE Publications; 1994.
45. Veldsman, M., et al. Neurorehab Neural Re, 2017. 31(2): p. 157-67.
46. Wilson, S.J., et al. *Cognitive models of music psychology and the lateralization of musical function within the brain.* Australian Journal of Psychology, 1999. **51**(3): p. 125-39.
47. Lee, Y.S., et al. *Effects of skill training on working memory capacity.* Learning and Instruction, 2007. **17**(3): 336-44.
48. Cheung, M.C., et al. *Music training is associated with cortical synchronization reflected in EEG coherence during verbal memory encoding.* PLoS ONE, 2017. **12**(3): e0174906.
49. Degé, F., et al. *Music lessons and intelligence: A relation mediated by executive functions.* Music Perception, 2011. **29**(2): 195-201.
50. Merrett, D.L., and Wilson, S.J. *Music and neural plasticity.* In N.S. Rickard & K. McFerran (Eds.), Lifelong engagement with music, 2012. New York: Nova Science Publishers Inc: pp. 119-61.
51. Wan, C.Y., and Schlaug, G. *Music making as a tool for promoting brain plasticity across the life span*. The Neuroscientist, 2010. *16*(5): 566-77.
