# Supplementary material for: Poststroke Cardiorespiratory Exercise for Brain Volume and Cognition: A Randomized Clinical Trial
Source: JAMA Netw Open. 2025 Aug 26;8(8):e2528907. doi: 10.1001/jamanetworkopen.2025.28907 (PMC12381666; doi:10.1001/jamanetworkopen.2025.28907)
Supplement: Supplement 2. — eTable 1. Per-Protocol Included Participants: Baseline Demographics, Relevant Brain Volumes, Vascular Risk Factors, and Cognitive Tests eTable 2. Summary of Exercise Intervention Per-Protocol Included Participants eTable 3. Summary of Primary, Secondary, and Exploratory Outcomes Per-Protocol Analysis eTable 4. Adverse Events Categorized by Organ Type During and Post Intervention eTable 5. Serious Adverse Events Categorized by Organ Type During and Post Intervention eFigure 1. Sensitivity Analysis for Change in Hippocampal Volume From 2 to 4 Months Modified Intention-to-Treat Population eFigure 2. Sensitivity Analysis for Change in Hippocampal Volume From 2 to 4 Months Per-Protocol Population eFigure 3. Forest Plots for Prespecified Subgroup Analyses for Per-Protocol Population eFigure 4. Illustration of Treatment Protocol and Progressive Overload eFigure 5. Stroke Lesion Overlap Map for Participants in the CON and CRX Groups eAppendix 1. MRI Scanner and (T1-Weighted MPRAGE) Acquisition Details eAppendix 2. MRI Original Sequences [file jamanetwopen-e2528907-s002.pdf]

Supplementary Online Content

Brodtmann A, Churilov L, Adkins K, et al; PISCES-ZODIAC Investigators. Poststroke cardiorespiratory exercise for brain volume and cognition: a randomized clinical trial. *JAMA Netw Open.* 2025;8(8):e2528907. doi:10.1001/jamanetworkopen.2025.28907

eTable 1. Per-Protocol Included Participants: Baseline Demographics, Relevant Brain Volumes, Vascular Risk Factors, and Cognitive Tests
eTable 2. Summary of Exercise Intervention Per-Protocol Included Participants
eTable 3. Summary of Primary, Secondary, and Exploratory Outcomes Per-Protocol Analysis
eTable 4. Adverse Events Categorized by Organ Type During and Post Intervention
eTable 5. Serious Adverse Events Categorized by Organ Type During and Post Intervention
eTable 5. Serious Adverse Events Categorized by Organ Type During and Post Intervention
eFigure 1. Sensitivity Analysis for Change in Hippocampal Volume From 2 to 4 Months Modified Intention-to-Treat Population
eFigure 2. Sensitivity Analysis for Change in Hippocampal Volume From 2 to 4 Months Per-Protocol Population
eFigure 3. Forest Plots for Prespecified Subgroup Analyses for Per-Protocol Population
eFigure 4. Illustration of Treatment Protocol and Progressive Overload
eFigure 5. Stroke Lesion Overlap Map for Participants in the CON and CRX Groups
eAppendix 1. MRI Scanner and (T1-Weighted MPRAGE) Acquisition Details
eAppendix 2. MRI Original Sequences

This supplementary material has been provided by the authors to give readers additional information about their work.

© 2025 Brodtmann A et al. *JAMA Network Open.*

Supplementary Tables

eTable 1. Per-Protocol Included Participants: Baseline Demographics, Relevant Brain Volumes, Vascular Risk Factors, and Cognitive Tests

	**CRX arm (n=41)**	**CON arm (n=51)**
**Demographics (baseline)**		
Age in years, median (IQR)	68·90 (55·81, 73·63)	67·47 (57·56, 75·76)
Assigned sex at birth[$], n (%)		
Male	23 (56·10%)	36 (70·59%)
Female	18 (43·90%)	15 (29·41%)
Years of Education, median (IQR)	15 (12, 17)	15 (12, 17)
Cardiorespiratory fitness[#], median (IQR)	15·4 (12·2, 23·4) n=40	16·6 (13·6, 24·2) n=51
Modified Rankin Scale, median (IQR)	1 (1, 2)	1 (1, 2)
National Institute of Health Stroke Scale*, median (IQR)	0 (0, 1) n=41	0·5 (0, 1) n=50
First ever clinical stroke, n (%)	38 (92·68%)	39 (76·47%)
**Brain MRI**		
Baseline hippocampal volume in mm^3, mean, (SD)	3600·40 (419·91)	3609·91 (449·32)
Baseline total brain volume in mm^3, mean (SD)	1121747 (125529·6)	1124009 (124842·7)
Baseline stroke lesion volume in mm^3, median (IQR)	1706 (280, 6305)	1308 (418, 4294)
**Vascular Risk Factors (n, %)**		
Atrial fibrillation, n (%)	3 (7·89%) n=38	13 (27·66%) n=47
Hypertension, n (%)	20 (50·00%) n=40	30 (58·82%) n=51
Dyslipidaemia, n (%)	20 (48·78%)	32 (62·75%)
Obesity^, n (%)	8 (20·51%) n=39	13 (27·08%) n=48
Type 2 Diabetes Mellitus, n (%)	7 (18·42%) n=38	5 (11·11%) n=45
Smoking history[@], n (%)	16 (39·02%)	27 (52·94%)
**Cognitive status at baseline**		
National Adult Reading Test, mean (SD)	108·72 (10·90) n=40	109·85 (9·43) n=49
Montreal Cognitive Assessment, median (IQR)	26 (24, 27)	25 (23, 27)
Trail Making Test – B, n (%)	41 (100%)	51 (100%)
Time to completion TMT-B in seconds, median (IQR)	85 (58, 125·1)	82·7 (68, 138)

© 2025 Brodtmann A et al. *JAMA Network Open.*

	n=41	n=50

CRX=cardiorespiratory; CON=control.
Note: Baseline=t_1, study entry; $all participants identified as cisgender to their assigned sex at birth; #cardiorespiratory fitness as measured by modified heart rate; *National Institute of Health Stroke Scale done at baseline assessment; ^BMI>30; @smoking status reported as never smoked versus history of smoking/current smoker for our analyses.

eTable 2. Summary of Exercise Intervention Per-Protocol Included Participants

	**CRX arm (n=41)**	**CON arm (n=51)**
Total number of exercise sessions per participant, median (IQR)	21 (20, 23)	21 (20, 24)
Total time spent in minutes spent with Trained Exercise professional per participant, median (IQR)	1200 (1098, 1380)	1235 (1199, 1440)
Mean time spent in cardiorespiratory exercise training per completed sessions per participant in minutes, median (IQR)	25·48 (24·4, 25·9)	N/A
Total time spent in cardiorespiratory exercise training per participant in minutes, median (IQR)	521 (497, 583)	N/A

© 2025 Brodtmann A et al. *JAMA Network Open.*

eTable 3. Summary of Primary, Secondary, and Exploratory Outcomes Per-Protocol Analysis

	**CRX arm (n=41)**	**CON arm (n=51)**	**Effect Size (95% CI)**	**P-value**
**Primary Outcome**				
Hippocampal volume in mm^3 at 4 months post-stroke, mean (SD)	3605·63 (395·48)	3610·53 (458·06)		
Hippocampal volume change between 2- and 4-months post-stroke, mean(SD)	-0·0025 (0·022)	-0·00024 (0·023)	-0·0025 (-0·012, 0·0067)	0·60
**Secondary Efficacy Outcomes**				
Total Brain Volume in mm^3 at 4-months post-stroke, mean (SD)	1119576 (125413·6)	1121314 (124494·6)		
Total Brain Volume change between 2- and 4-months post-stroke, mean (SD)	0·0018 (0·011)	0·0023 (0·011)	-0·00040 (-0·0049, 0·0041)	
12-month Number/proportion TMT-B, n (%)	37 (90·24%)	48 (94·12%)		
12-month TMT-B, median (IQR)	91 (58, 113·2) n=37	88·4 (59, 112) n=48	-6·93 (-11·12, -2·73)	
**Safety Outcomes intervention period 2-4 months post-stroke**				
Death, n (%)	0 (0%)	0 (0%)	N/E	
SAE, n (%)	2 (4·88%)	3 (5·88%)	0·75 (0·14, 4·09)[1]	
Recurrent stroke/TIA, n (%)	0 (0%)	1 (1·96%)	-0·020 (-0·058, 0·018)[2]	
**Exploratory Imaging outcomes**				
Hippocampal volume at 12 months in mm^3, mean (SD)	3561·62 (434·30) n=36	3606·10 (447·86) n=46		
Hippocampal volume change between 2- and 12-months post-stroke, mean (SD)	0·0092 (0·025) n=36	0·011 (0·021) n=46	-0·0012 (-0·011, 0·0085)	
Total Brain Volume at 12 months in mm^3, mean (SD)	1120884 (121927·4)	1125766 (118544·6)		

© 2025 Brodtmann A et al. *JAMA Network Open.*

	n=36	n=46		
Total Brain Volume change between 2- and 12-months post-stroke, mean (SD)	0·0062 (0·012)<br>n=36	0·010 (0·014)<br>n=46	-0·0040<br>(-0·010, 0·0023)	
**Exploratory Outcomes measured at 12 months**				
Cardiorespiratory Fitness as$V0_{2peak,}$ estimate, mean (SD)	16·68 (5·28)<br>n=33	18·67 (5·27)<br>n=43	-0·28<br>(-1·59, 1·02)	
Number of participants with ADAS-Cog, n (%)	28 (68·29%)	29 (56·86%)		
ADAS-Cog score, median (IQR)	8·67 (6·67, 13·5)<br>n=28	10·33 (8·67, 14·33)<br>n=29	-1·33<br>(-1·69, -0·98)	
Modified Rankin Scale, n (%)				
mRS 0<br>mRS 1<br>mRS 2<br>mRS 3<br>mRS 4<br>mRS 5<br>mRS 6	6 (16·22%)<br>23 (62·16%)<br>6 (16·22%)<br>2 (5·41%)<br>0 (0%)<br>0 (0%)<br>0 (0%)<br>n=37	11 (22·92%)<br>30 (62·50%)<br>7 (14·58%)<br>0 (0%)<br>0 (0%)<br>0 (0%)<br>0 (0%)<br>n=48	0·55<br>(0·23, 1·33)	
PASE Daily Physical Activity, median (IQR)	160·23 (95·8, 185·45)<br>n=34	162·67 (78·36, 199·9)<br>n=46	2·55<br>(-34·43, 39·53)	
Anxiety GAD-7 score, median (IQR)	2 (0, 4)<br>n=33	1 (0, 3)<br>n=46	0<br>(-0·43, 0·43)	
Depression PHQ-9, median (IQR)	5 (2, 8)<br>n=33	2 (1, 5)<br>n=46	1 (0·11, 1·89)	
Quality of Life AQoL, median (IQR)	88·89 (80·56, 94·44)<br>n=34	91·67 (86·11, 94·44)<br>n=46	-0·93<br>(-1·46, -0·39)	

© 2025 Brodtmann A et al. *JAMA Network Open.*

Fatigue FAS, median (IQR)	22 (17, 26)<br>n=33	19 (16, 22·5)<br>n=44	-1·31<br>(-6·00, 3·38)	

PASE=physical activity scale for the elderly; GAD-7=generalized anxiety scale-7; PHQ-9=patient health questionnaire-9; AQoL=assessment of quality of life; FAS=fatigue assessment scale

Note, due to only a small number of safety events effect sizes were calculated as: [1]odds ratio using Firth logistic regression; [2]unadjusted risk difference; N/E non-estimable due to no events

© 2025 Brodtmann A et al. *JAMA Network Open.*

eTable 4. Adverse Events Categorized by Organ Type During and Post Intervention

	**CRX Arm**		**CON Arm**	
	During Intervention (n=32)	Post Intervention (n=20)	During Intervention (n=34)	Post Intervention (n=17)
Cardiovascular^	8	6	1	4
Dermatological*		3	1	1
Respiratory				
Recurrent stroke or TIA				
Musculoskeletal	11	2	7	6
Trips or falls	3	1	12	
Infection	1	2		
Neurological[a]	4	2	11	2
Psychiatric				1
Endocrine		1		
Gastrointestinal				
Cancer		1		
SARS-CoV-2		1	1	
Study equipment fault[o]	1			3
Other	4	1	1	

Note: ^Excluding stroke/TIA i·e·, arrhythmia's, atrial fibrillation, chest pain. *Excluding abrasions from devices, equipment) i·e· eczema, abrasions or cuts to the skin. [a]Other than TIA/recurrent stroke i·e·, dizziness or vertigo, limb numbness or tingling. [o]I·e·, skin abrasion due to card Xplore machine, reaction to heart rate monitoring equipment during intervention

eTable 5. Serious Adverse Events Categorized by Organ Type During and Post Intervention

	**CRX Arm**		**CON Arm**	
	During Intervention (n=3)	Post Intervention (n=4)	During Intervention (n=3)	Post Intervention (n=4)
Cardiovascular^	1	1		
Dermatological*				
Respiratory	1	1	1	
Recurrent stroke or TIA				
Musculoskeletal				
Trips or falls	1			1
Infection		1	1	2
Neurological[a]		1		
Psychiatric				
Endocrine				
Gastrointestinal				1
Cancer				
SARS-CoV-2				
Study equipment fault[o]			1	
Other				

Note: ^Excluding stroke/TIA i·e·, arrhythmia's, atrial fibrillation, chest pain. *Excluding abrasions from devices, equipment) i·e· eczema, abrasions or cuts to the skin. [a]Other than TIA/recurrent stroke

© 2025 Brodtmann A et al. *JAMA Network Open.*

i·e·, dizziness or vertigo, limb numbness or tingling. ºI·e·, skin abrasion due to card Xplore machine, reaction to heart rate monitoring equipment during intervention

© 2025 Brodtmann A et al. *JAMA Network Open.*

Supplementary Figures

eFigure 1. Sensitivity Analysis for Change in Hippocampal Volume From 2 to 4 Months Modified Intention-to-Treat Population

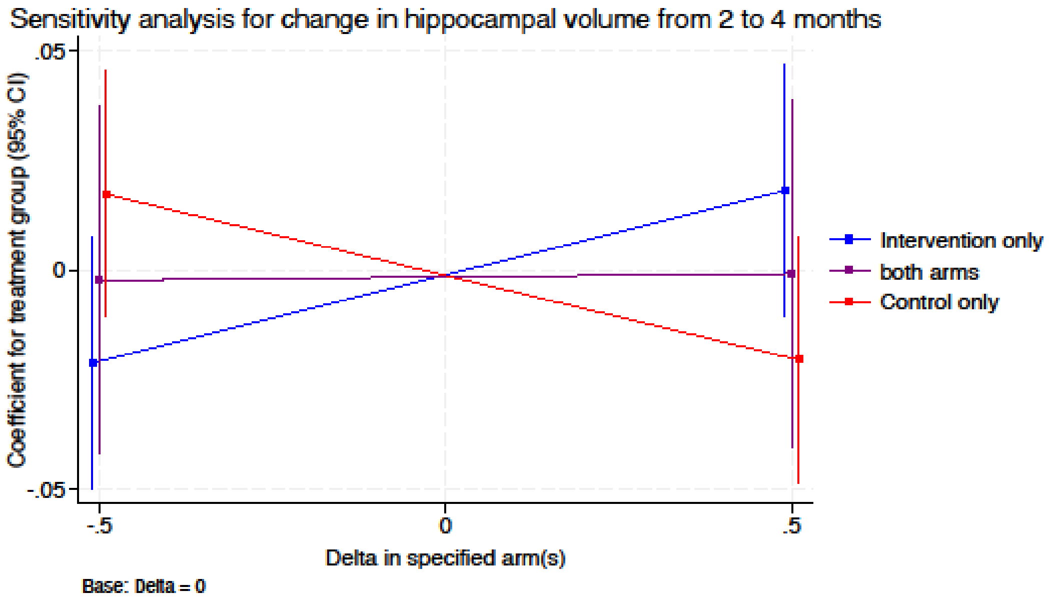

Note, Base: Delta = 0. Click here to open image document.

eFigure 2. Sensitivity Analysis for Change in Hippocampal Volume From 2 to 4 Months Per-Protocol Population

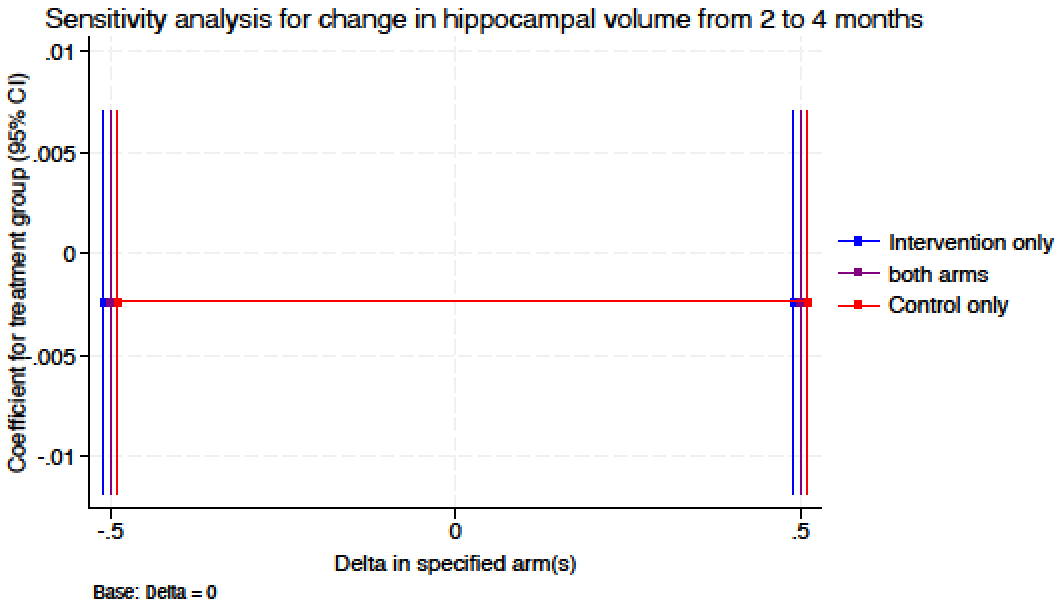

Note, Base: Delta = 0. Click here to open image document.

© 2025 Brodtmann A et al. *JAMA Network Open.*

eFigure 3. Forest Plots for Prespecified Subgroup Analyses for Per-Protocol Population

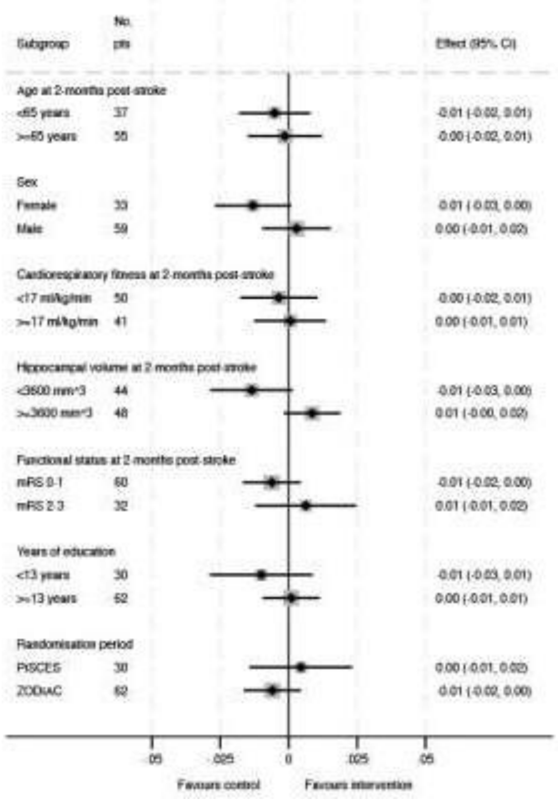

Note: Click here to open image document.

© 2025 Brodtmann A et al. *JAMA Network Open.*

eFigure 4. Illustration of Treatment Protocol and Progressive Overload

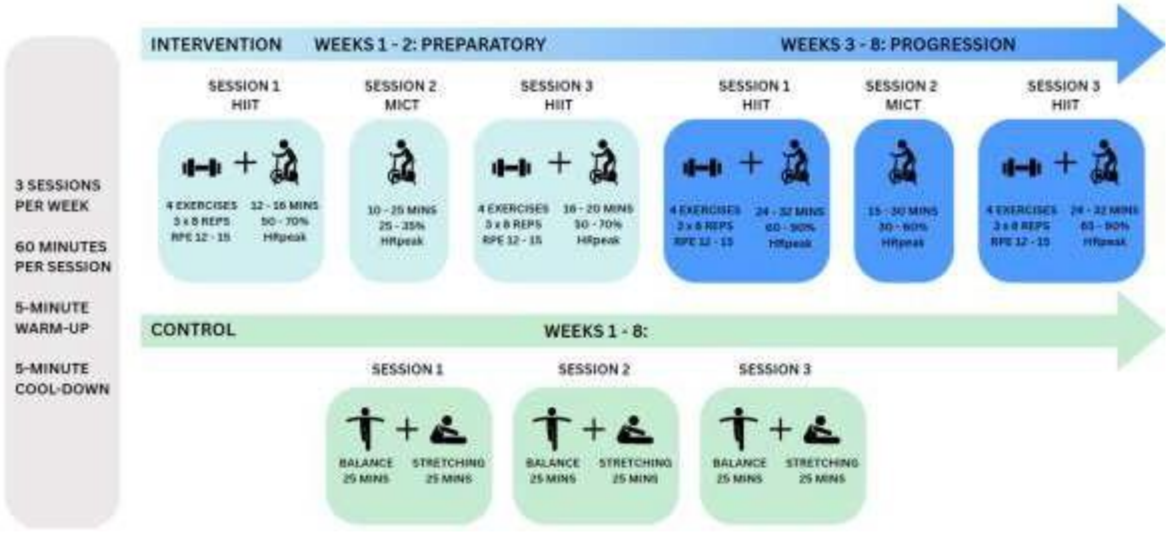

HIIT, High Intensity Interval Training; MICT, Moderate Intensity Continuous Training; HRpeak, peak heart rate recorded during baseline fitness testing; RPE, Rating of Perceived Exertion (6-20 scale; Borg, 1960).

Resistance training exercises targeted major muscle groups including the shoulders, pectorals, triceps, biceps, trunk, gluteals, quadriceps, and calves.

Stretching exercises focussed on the major muscle groups including the shoulders, triceps, back, gluteals, hamstrings, quadriceps, and calves. Balance exercises included functional reach up/down, heel-toe walking, single-leg standing, self-paced stepping, and agility walking.

Note: Click here to open image document.

© 2025 Brodtmann A et al. *JAMA Network Open.*

eFigure 5. Stroke Lesion Overlap Map for Participants in the CON and CRX Groups

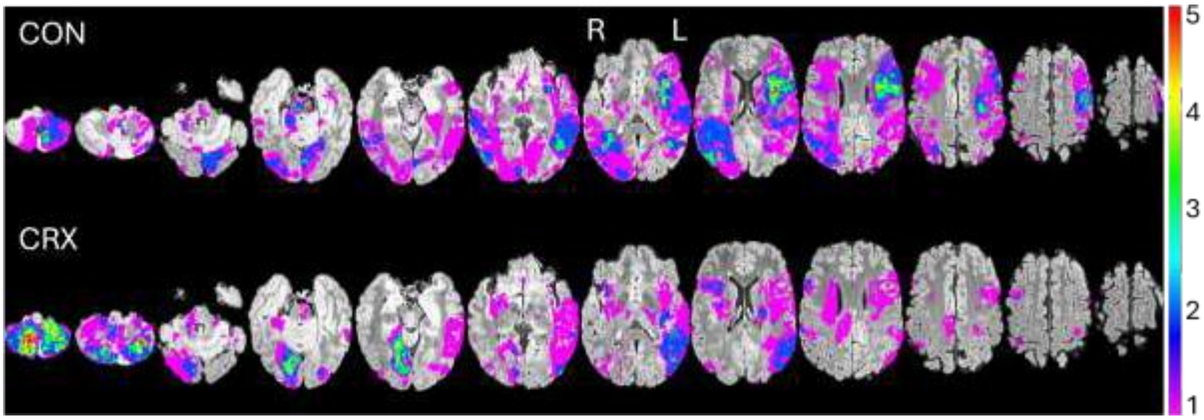

Colours indicate the number of participants with infarction at each voxel (red = highest overlap). The group-level overlap maps were generated from binarized manual lesions delineated on T2-FLAIR images, registered to a common image in the cohort, then superimposed using FSL/fslmaths.

Note: click here to open image document.

© 2025 Brodtmann A et al. *JAMA Network Open.*

eAppendix 1. MRI Scanner and (T1-Weighted MPRAGE) Acquisition Details

Details from three recruitment/scanning sites (Baker Institute, The Alfred; Melbourne Brain Centre, The Florey; Sunshine Hospital, Western Health).

Site: Melbourne Brain Centre (MBC) | Scanner: Prisma


```
    Modality: MR,
    MagneticFieldStrength: 3,
    ImagingFrequency: 123.237901,
    Manufacturer: Siemens,
    ManufacturersModelName: Prisma_fit,
    InstitutionName: Melbourne Brain Centre,
    InstitutionalDepartmentName: Department,
    InstitutionAddress: Burgundy 345,Heidelberg,Victoria,AU,3084,
    DeviceSerialNumber: 167062,
    StationName: MRC35199,
    BodyPart: BRAIN,
    PatientPosition: HFS,
    ProcedureStepDescription: 2019_MBC_PROTOCOLS_VE11C^SB.2022.384 PISCES study - 64 channel,
    SoftwareVersions: syngo MR E11,
    MRAcquisitionType: 3D,
    StudyDescription: 2019_MBC_PROTOCOLS_VE11C^SB.2022.384 PISCES study - 64 channel,
    SeriesDescription: t1_mprage_sag_p2_iso_1mm stealth,
    ProtocolName: t1_mprage_sag_p2_iso_1mm stealth,
    ScanningSequence: GR\\IR,
    SequenceVariant: SK\\SP\\MP,
    ScanOptions: IR,
    SequenceName: *tfl3d1_16ns,
    ImageType: [ORIGINAL, PRIMARY, M, ND, NORM, MAGNITUDE],
    NonlinearGradientCorrection: false,
    SeriesNumber: 3,
    AcquisitionTime: 10:26:47.755000,
    AcquisitionNumber: 1,
    SliceThickness: 1,
    SAR: 0.0748001,
    TablePosition: [
        0,
        0,
        -0    ],
    EchoTime: 0.00243,
    RepetitionTime: 1.9,
    SpoilingState: true,
    InversionTime: 0.9,
    FlipAngle: 9,
    PartialFourier: 1,
    BaseResolution: 256,
    ShimSetting: [
        -1278,
        4846,
        -11270,
        38,
        33,
        -42,
        -49,
        7     ],
    TxRefAmp: 243.073,
    PhaseResolution: 1,
    ReceiveCoilName: HeadNeck_64,
    ReceiveCoilActiveElements: HC1-7,
    PulseSequenceDetails: %SiemensSeq%\\tfl,
    RefLinesPE: 24,
    CoilCombinationMethod: Adaptive Combine,
    ConsistencyInfo: N4_VE11C_LATEST_20160120,
    MatrixCoilMode: GRAPPA,
    PercentPhaseFOV: 100,
    PercentSampling: 100,
    PhaseEncodingSteps: 255,
    AcquisitionMatrixPE: 256,
    ReconMatrixPE: 256,
    ParallelReductionFactorInPlane: 2,
    PixelBandwidth: 180,
    DwellTime: 1.09e-05,
    ImageOrientationPatientDICOM: [
        -0.0749787,
        0.997185,
        -2.59076e-08,
        -0.0330619,
        -0.00248596,
        -0.99945      ],
    ImageOrientationText: Sag>Cor(-4.3)>Tra(1.9),
    InPlanePhaseEncodingDirectionDICOM: ROW,
  BidsGuess: [anat,_acq-tfl3p2_run-3_T1w],
```


Note: Double click here to open image document.

© 2025 Brodtmann A et al. *JAMA Network Open.*

eAppendix 2. MRI Original Sequences

Original (highlighted) MRI sequences and their derivatives acquired at the Melbourne Brain Centre, The Florey Institute (sequences were replicated at other sites

1 localiser
2 T2_spc_sag_p2_iso_1.0
3 t1_mprage_sag_p2_iso_1mm stealth
4 t2_tirm_spc_da-fl_sag_p2_iso_1.0
5 3T_fl3d_tra_p2_swi_FA15 0p5_Mag_Images
6 3T_fl3d_tra_p2_swi_FA15 0p5_Pha_Images
7 3T_fl3d_tra_p2_swi_FA15 0p5_mIP_Images(SW)
8 3T_fl3d_tra_p2_swi_FA15 0p5_SWI_Images
9 PRE DWI A-P DIST CORR
10 PRE DWI P-A DIST Change 180
11 FREE DWI 71 directions ep2d_diff_mbc_p2
12 FREE DWI 71 directions ep2d_diff_mbc_p2_ADC
13 FREE DWI 71 directions ep2d_diff_mbc_p2_TRACEW
14 FREE DWI 71 directions ep2d_diff_mbc_p2_FA
15 FREE DWI 71 directions ep2d_diff_mbc_p2_ColFA
16 FREE DWI 71 directions ep2d_diff_mbc_p2_TENSOR
17 gre_field_mapping +30 DEG
18 gre_field_mapping +30 DEG
19 FREE RUN ep2d_bold_moco_p2 7MIN
20 FREE RUN ep2d_bold_moco_p2 7MIN_StartFMRI
21 FREE RUN ep2d_bold_moco_p2 7MIN_MoCoSeries
22 FREE RUN ep2d_bold_moco_p2 7MIN_intermediate t-Map
23 FREE RUN ep2d_bold_moco_p2 7MIN_Design
24 FREE RUN ep2d_bold_moco_p2 7MIN_EvaSeries_GLM
25 FREE RUN ep2d_bold_moco_p2 7MIN_Mean_&_t-Maps
26 FREE RUN ep2d_bold_moco_p2 7MIN
27 FREE RUN ep2d_bold_moco_p2 7MIN_StartFMRI
28 FREE RUN ep2d_bold_moco_p2 7MIN_MoCoSeries
29 FREE RUN ep2d_bold_moco_p2 7MIN_intermediate t-Map
30 FREE RUN ep2d_bold_moco_p2 7MIN_Design
31 FREE RUN ep2d_bold_moco_p2 7MIN_EvaSeries_GLM
32 FREE RUN ep2d_bold_moco_p2 7MIN_Mean_&_t-Maps
33 3T_fl3d_tra_p2_swi_FA15 0p5_RR
34 3T_fl3d_tra_p2_swi_FA15 0p5_RR
35 Phoenix Document_PhoenixZIPReport
36 T2_spc_sag_p2_iso_1.0_T2 AX 2MM MPR
37 T2_spc_sag_p2_iso_1.0_T2 COR 2MM MPR
38 T2_spc_sag_p2_iso_1.0_T2 SAG 2MM MPR
39 t2_tirm_spc_da-fl_sag_p2_iso_1.0_T2 FLAIR AX 2MM MPR
40 t2_tirm_spc_da-fl_sag_p2_iso_1.0_T2 FLAIR COR 2MM MPR
41 t2_tirm_spc_da-fl_sag_p2_iso_1.0_T2 FLAIR SAG 2MM MPR
42 t1_mprage_sag_p2_iso_1mm stealth_T1 AX 2MM MPR
43 t1_mprage_sag_p2_iso_1mm stealth_T1 COR 2MM MPR
44 t1_mprage_sag_p2_iso_1mm stealth_T1 SAG 2MM MPR

Note: Double click here to open image document.

© 2025 Brodtmann A et al. *JAMA Network Open.*
