# Supplementary material for: Poststroke Cardiorespiratory Exercise for Brain Volume and Cognition: A Randomized Clinical Trial
Source: JAMA Netw Open. 2025 Aug 26;8(8):e2528907. doi: 10.1001/jamanetworkopen.2025.28907 (PMC12381666; doi:10.1001/jamanetworkopen.2025.28907)
Supplement: Supplement 3. — Nonauthor Collaborators [file jamanetwopen-e2528907-s003.pdf]

Supplemental Online Content: Nonauthor Collaborators
*First name, last name, and suffix (if applicable) are required and will appear in PubMed.

***Group Name(s): PISCES-ZODIAC Investigators**							
***First Name and Middle Initial(s)**	***Last Name**	***Suffix (eg, Jr, III)**	Academic Degrees	Institution	Location (city, state/province, country)	Role or Contribution, eg, chair, principal investigator	Group (if more than 1 Group listed in the byline) and/or Subgroup (eg, Steering Committee)
Rachel	Ellis		PhD	Monash University	Melbourne, Victoria, Australia	Project Staff	N/A
Laura	Bird		PhD	Monash University	Melbourne, Victoria, Australia	Project Staff	N/A
Chris	Shirbin		PhD	Royal Prince Alfred Hospital Network	Sydney, NSW, Australia	Project Staff	N/A
Elizabeth	McInerney		BHSc-Hns	Monash University	Melbourne, Victoria, Australia	Project Staff	N/A
Laura	White		MSc	Monash University	Melbourne, Victoria, Australia	Project Staff	N/A
Alex	Billett		BPsychSc-Hns	Monash University	Melbourne, Victoria, Australia	Project Staff	N/A
Deena	Ebaid		PhD	Rare Disease Team Ipsen	Melbourne, Victoria, Australia	Project Staff	N/A
Antonia	Clarke		MD	Monash University	Melbourne, Victoria, Australia	Randomisation Schedule Holder	N/A
Elie	Gottlieb		PhD	SleepScore Labs	Los Angeles, California, Australia	Randomisation Schedule Holder	N/A
Christopher	Bladin		MD	Monash University	Melbourne, Victoria, Australia	Independent Medical Monitor	N/A
Francine	Marques		PhD	Monash University	Melbourne, Victoria, Australia	Microbiome Projects analyses	N/A
Phillip	Choi		PhD	Monash University	Melbourne, Victoria, Australia	Eastern Health site contact	N/A
Tanya	Frost		MAdvN	Eastern Health	Melbourne, Victoria, Australia	Eastern Health site contact	N/A
Matthew	Wingfield		MPhys	Epworth Healthcare	Melbourne, Victoria, Australia	Epworth Healthcare site contact	N/A
Tissa	Wijeratne		PhD	Western health	Melbourne, Victoria, Australia	Western Health site contact	N/A
Sherisse	Celestino		BPsychSc(Hons)	Western Health	Melbourne, Victoria, Australia	Western Health site contact	N/A
Toby	Cumming		PhD	Monash University	Melbourne, Victoria, Australia	Project Consultant	N/A
Vladimir	Hachinski		PhD	University of Western Ontario	London, Ontario, Canada	Project Consultant	N/A
Nicola	Lautenschlager		PhD	University of Melbourne	Melbourne, Victoria, Australia	Project Consultant	N/A
David	Darby		PhD	Monash University	Melbourne, Victoria, Australia	Project Consultant	N/A
