# Supplementary material for: Poststroke Cardiorespiratory Exercise for Brain Volume and Cognition: A Randomized Clinical Trial
Source: JAMA Netw Open. 2025 Aug 26;8(8):e2528907. doi: 10.1001/jamanetworkopen.2025.28907 (PMC12381666; doi:10.1001/jamanetworkopen.2025.28907)
Supplement: Supplement 4. — Data Sharing Statement [file jamanetwopen-e2528907-s004.pdf]

Data Sharing Statement

Brodtmann. Poststroke Cardiorespiratory Exercise for Brain Volume and Cognition. *JAMA Netw Open*. Published August 26, 2025. doi:10.1001/jamanetworkopen.2025.28907

Data
Additional Information: ANZCTR ACTRN12616000942459
https://anzctr.org.au/Trial/Registration/TrialReview.aspx?ACTRN=ACTRN12616000942459
Data available: Yes
Data types: Deidentified participant data, Data dictionary
How to access data: These data will be shared upon request after submission and review to our Data Steering Committee. Contact amy.brodtmann@monash.edu
When available: beginning date: 01-01-2027

Supporting Documents
Document types: None

Additional Information
Who can access the data: Data will be made available to approved researchers whose proposed use of the data has been approved by the Steering Committee.
Types of analyses: Data will be made available for prespecified purposes only.
Mechanisms of data availability: After approval and with signed data access agreement.
